# Autoimmune and neuropsychiatric phenotypes in a *Mecp2* transgenic mouse model on C57BL/6 background

**DOI:** 10.3389/fimmu.2024.1370254

**Published:** 2024-03-08

**Authors:** Yaxi Li, Shu Zhang, Chenling Tang, Bowen Yang, Fatin Atrooz, Zhifeng Ren, Chandra Mohan, Samina Salim, Tianfu Wu

**Affiliations:** ^1^ Department of Biomedical Engineering, University of Houston, Houston, TX, United States; ^2^ Department of Pharmacological and Pharmaceutical Sciences, College of Pharmacy, University of Houston, Houston, TX, United States; ^3^ Department of Physics, University of Houston, Houston, TX, United States

**Keywords:** NPSLE, mouse model, MeCP2, autoantigen array, immune phenotypes, behavior tests

## Abstract

**Introduction:**

Systemic Lupus Erythematosus (SLE) impacts the central nervous system (CNS), leading to severe neurological and psychiatric manifestations known as neuropsychiatric lupus (NPSLE). The complexity and heterogeneity of clinical presentations of NPSLE impede direct investigation of disease etiology in patients. The limitations of existing mouse models developed for NPSLE obstruct a comprehensive understanding of this disease. Hence, the identification of a robust mouse model of NPSLE is desirable.

**Methods:**

C57BL/6 mice transgenic for human MeCP2 (B6.*Mecp2^Tg1^*) were phenotyped, including autoantibody profiling through antigen array, analysis of cellularity and activation of splenic immune cells through flow cytometry, and measurement of proteinuria. Behavioral tests were conducted to explore their neuropsychiatric functions. Immunofluorescence analyses were used to reveal altered neurogenesis and brain inflammation. Various signaling molecules implicated in lupus pathogenesis were examined using western blotting.

**Results:**

B6.*Mecp2^Tg1^* exhibits elevated proteinuria and an overall increase in autoantibodies, particularly in female B6.*Mecp2^Tg1^* mice. An increase in CD3^+^CD4^+^ T cells in the transgenic mice was observed, along with activated germinal center cells and activated CD11b^+^F4/80^+^ macrophages. Moreover, the transgenic mice displayed reduced locomotor activity, heightened anxiety and depression, and impaired short-term memory. Immunofluorescence analysis revealed IgG deposition and immune cell infiltration in the kidneys and brains of transgenic mice, as well as altered neurogenesis, activated microglia, and compromised blood-brain barrier (BBB). Additionally, protein levels of various key signaling molecules were found to be differentially modulated upon MeCP2 overexpression, including GFAP, BDNF, Albumin, NCoR1, mTOR, and NLRP3.

**Discussion:**

Collectively, this work demonstrates that B6.*Mecp2^Tg1^* mice exhibit lupus-like phenotypes as well as robust CNS dysfunctions, suggesting its utility as a new animal model for NPSLE.

## 1 Introduction

Involvement of the nervous system in Systemic Lupus Erythematosus (SLE) gives rise to nonspecific and heterogeneous neuropsychiatric manifestations, and its mortality rate is surpassed only by that of Lupus Nephritis (LN) (1). Approximately 12%-95% of lupus patients present clinical CNS-related symptoms, encompassing headaches, cognitive impairments, and psychiatric disorders, collectively termed as Neuropsychiatric SLE (NPSLE). Nevertheless, the intricate mechanisms underpinning the pathogenesis of NPSLE remain obscure (2–4). Hence, it is critical to identify a suitable animal model that best resembles human NPSLE, thereby facilitating an in-depth exploration of NPSLE’s pathogenesis. This pursuit is pivotal for a more profound comprehension of the underlying mechanisms and for the advancement of innovative diagnostic or therapeutic methodology catering to NPSLE patients.

To date, several lupus-prone mouse models displaying CNS manifestations have been employed for investigating NPSLE, exemplified by the MRL/MpJ-*Fas* ^lpr/lpr^ (MRL/*lpr*) (5–7). However, a recent study has indicated that the neuropsychiatric manifestation of MRL/*lpr* mice is confined primarily to depression-like behavior, without discernible distinctions in other behavioral assessments encompassing general activity, motor coordination, anxiety-like behavior, cognition activity as well as social ability (5). The lack of robust behavioral phenotypes within this mouse strain restricts its viability as a definitive NPSLE mouse model. Inquiries into Neuropsychiatric SLE have also encompassed the use of New Zealand black/New Zealand white (NZB/W) F1 and BXSB mice which have also been used in NPSLE studies; nonetheless, both NZB/W F1 and BXSB mice exhibit an even lower incidence of behavioral phenotypes compared to MRL/*lpr* mice (8, 9). Additionally, B6.*lpr* mice and B6.*Sle1.Sle3* strains have displayed certain levels of depression-like and anxiety-like behaviors (10, 11). Nevertheless, the attenuated lupus phenotypes and delayed disease onset in B6.*lpr* and B6.*Sle1.Sle3* mice restrain their application for the NPSLE study. Given these multifaceted challenges, there is an urgent need to identify a new robust mouse model for NPSLE, thereby enabling an in-depth exploration of the underlying disease mechanisms.

Methyl-CpG binding protein 2 (MeCP2), a protein encoded by a gene located on chromosome Xq28, selectively binds 5-methyl cytosine residues in CpG dinucleotides in mammalian genomes (12, 13). Intriguingly, the expression level of MeCP2 has been established as a pivotal contributor in neurological diseases, such as Rett syndrome (RTT) (14–17). A transgenic murine model was created through the insertion of a human *Mecp2* gene, resulting in the overexpression of the MeCP2 protein (18–20). This transgenic mouse model develops a progressive neurological phenotype, exhibiting stereotypy and repetitive movements, epilepsy, spasticity, hypoactivity, and early death (19).

More interestingly, *Mecp2* was identified as one of the susceptibility genes associated with SLE through a genome-wide association study (GWAS) (21–25). *Mecp2* transgenic mice bred on the FVB/N background exhibited elevated levels of anti-nuclear antibodies (26). Significantly, a recent study conducted by Yang et al. identified an impairment in the TH1 immune response and IFN-γ production in children presenting with postnatal neurological syndromes arising from *Mecp2* duplication. This group of patients also exhibited variable immunological abnormalities, including reductions in memory T and B cells, natural killer cells, and responses in immunoglobulin (Ig) assay (20).

We hypothesize that C57BL/6 mice transgenic for human *Mecp2* (designated as B6. *Mecp2^Tg1^*) could potentially exhibit concurrent autoimmune and neuropsychiatric-resembling characteristics. In this study, we undertook a comprehensive evaluation through the exploration of autoantibody profiles, immune cell subpopulations and their activation status, proteinuria levels, neuropsychiatric phenotypes, and pertinent signaling pathways. The objective was to ascertain the feasibility of using B6.*Mecp2^Tg1^* mouse model as a novel tool for investigating NPSLE.

## 2 Materials and methods

### 2.1 Animals

Six-week-old female C57BL/6J (B6, stock# 000664) mice and age-matched female MRL/MpJ-*Fas* ^lpr^/J (MRL*/lpr*, stock# 000485) mice were purchased from the Jackson Laboratory (Bar Harbor, ME). These mice were housed in the animal facility of the University of Houston (Houston, TX) and were afforded ad libitum access to food and water. Breeding pairs of mice transgenic for human *Mecp2* with a B6 background, hereafter referred to as B6.*Mecp2^Tg1^*, were generous gifts from Dr. Huda Zoghbi at the Baylor College of Medicine (Houston, TX). The B6.*Mecp2^Tg1^* strain comprises a 99-kb human PAC clone containing all exons of the *Mecp2* gene while lacking other transcriptional units for neighboring genes. This human-derived *Mecp2* gene insertion was presumed to have occurred within the X chromosome (19). B6.*Mecp2^Tg1^* mice were bred under specific pathogen-free (SPF) conditions, and all animal protocols were approved by the Institutional Animal Care and Use Committee (IACUC) at the University of Houston.

We studied adult female mice for both B6 and MRL/lpr strains, as well as both genders for the transgenic mice aged nine to seventeen weeks. Previous research on MRL/lpr indicated that female mice display more prominent signs of a lupus-like disease (27, 28). The B6.*Mecp2^Tg1^* strain exhibits abnormal behavior starting from 12 weeks of age (19), while MRL/lpr display NPSLE-like features at the same age (6).

### 2.2 Genotyping

Tail tissues were clipped from the litters and collected in RNase- and DNase-free microcentrifuge tubes. The collected tissues were subsequently treated with tail lysis buffer, containing 0.5% SDS, 0.1M NaCl, 0.05M Tris (pH 8.0), and 2.5 mM EDTA. Proteinase K (Catalog# AM2546, Thermo Fisher Scientific) was added into the pre-warmed lysis buffer at a concentration of 1 mg/ml prior to utilization. The primers, synthesized by Integrated DNA Technologies (IDT), were specifically designed for the amplification of the human-derived *Mecp2* gene by PCR: Forward primer, CGCTCTGCCCTATCTCTGA; Reverse primer, ACAGATCGGATAGAAGACTC.

### 2.3 Enzyme-linked immunosorbent assay

Serum samples obtained from all mice groups at different age intervals were tested for the presence of total immunoglobulins and anti-dsDNA antibodies using ELISA assays. In the case of total immunoglobulin detection, microplates were coated with 2 µg/ml goat anti-mouse IgG (Southern Biotech) or IgM. Following blocking, serially diluted immunoglobulin (Ig) standards (IgG, Catalog# I5381, Sigma-Aldrich; mouse IgM, catalog# 010-0107, Rockland) or serum samples were added into the microplate. After a two-hour incubation at room temperature, the bound Igs were detected with alkaline phosphatase-conjugated goat anti-mouse IgG (Catalog# 2040-04, Southern Biotech) or goat anti-mouse IgM (Catalog# 626822, Invitrogen). Subsequently, quantification was carried out by measuring the absorbance at 405 nm. For the detection of IgG anti-dsDNA antibodies, 100 μg/ml of mBSA in phosphate-buffered saline (PBS) was applied to pre-coat the plates at 37°C for thirty minutes prior to the incubation of dsDNA standards (Catalog# D1501, Sigma) with an initial concentration at 1250 ng/ml. Serum samples were diluted 1:50 in PBS and added to the plates for a two-hour incubation. Alkaline phosphatase-conjugated goat anti-mouse IgG was used as the detection antibody.

### 2.4 Flow cytometry

Spleens were harvested from all mice groups at 17 weeks of age, and Cell Staining Buffer (Catalog# 420201, BioLegend) was used for the preparation of single-cell suspensions. Following cell quantification using the Countess™ II Automated Cell Counter (Catalog#A27977, Thermo Fisher Scientific), splenic cells were adjusted to a concentration of 1 x 10^7^ cells per milliliter. Cell suspensions of 100 µl per well were added into a 96 well V-bottom plate (Catalog# 3894, Corning), each well containing 1 x 10^6^ suspended cells. A pre-incubation on ice for 10 minutes was conducted with 0.25 μg per well of TruStain FcX™ PLUS (anti-mouse CD16/32) (Catalog# 156604, BioLegend) to block non-specific immunoglobulin binding to Fc receptors.

Antibodies conjugated with FITC, PE, PE/Cy7, APC, and APC/Cy7 against CD3 (Clone 17A2, Catalog# 100204), CD4 (PE, Clone GK1.5, Catalog# 100408), CD4 (APC, Clone GK1.5, Catalog# 100412), CD8a (Clone 53-6.7, Catalog# 100712), CD69 (Clone H1.2F3, Catalog# 104526), CD44 (Clone IM7, Catalog# 103028), CD62L (Clone MEL-14, Catalog# 104418), CD25 (Clone 3C7, Catalog# 101904), B220 (Clone RA3-6B2, Catalog# 103222), CD19 (Clone 6D5, Catalog# 115506), CD21 (Clone 7E9, Catalog# 123408), CD23 (Clone B3B4, Catalog# 101608), CD86 (Clone GL-1, Catalog# 105012), GL7 (Clone GL7, Catalog# 144610), CD138 (Clone 281-2, Catalog# 142504), CD11b (Clone M1/70, Catalog# 101206), CD11c (Clone N418, Catalog# 117324), and F4/80 (Clone BM8, Catalog# 123110) were purchased from Biolegend. These conjugated antibodies were subsequently added to the plates following the designed panels for the identification of B cells, T cells, myeloid cells, and their respective subsets. Following a 20-minute incubation on ice in darkness, cells were washed twice with Cell Staining Buffer through centrifugation at 350 x g for five minutes, followed by the addition of 7-amino-actinomycin D (7-AAD) (Catalog# 420404, Biolegend) to discriminate dead cells. Analysis of the splenic single-cell suspensions was performed on a multicolor BD LSR II cell analyzer (BD Biosciences). FlowJo software was employed for data acquisition and subsequent analysis.

### 2.5 Antigen array

The detailed procedure for the antigen array has been described previously (29–31). A compilation of 85 putative autoantigens (**Supplementary Table 1**) identified in the literature was meticulously selected to establish the autoantigen array. These represent established autoantigens that have been studied in at least one of the autoimmune diseases, including SLE, rheumatoid arthritis (RA), Sjögren’s syndrome, or multiple sclerosis (MS) (30, 31).

The selected antigens were printed in a 2 × 8 block matrix layout onto epoxy-modified microarray slides (STRATEC Consumables). This was accomplished utilizing a microarray printing robot (sciFLEXARRAYER S3, Scienion) positioned within a controlled printing chamber at 25°C and 60% humidity. Serum samples derived from different mouse groups at 17 weeks were diluted with super G solution (Catalog# 105101, Grace Bio-Labs). Each subarray was exposed to 100 μl of the diluted serum sample for subsequent detection using Cy5-conjugated goat anti-mouse IgG (Catalog# 115-175-166, Jackson ImmunoResearch). The array slides were scanned using a GenePix Microarray Scanner 4400A (Molecular Devices LLC, USA), and each individual spot was assessed and quantified through GenePix Pro 7 software (Molecular Devices LLC, USA). A heatmap depicting the antigen array was generated and evaluated employing RStudio (Version 1.3.959; RStudio, Inc.) utilizing average linkage Euclidean distance hierarchical clustering.

### 2.6 Behavioral testing

All tests as detailed below were conducted under low incandescent lighting conditions and were digitally documented by ANY-maze software (Stoelting Co., IL, USA). Behavioral tests were conducted three times across development, at 9-, 13-, and 17-week-old. Subsequent measurement of all data were executed employing ANY-maze software and subjected to further analysis via ImageJ (NIH) software (http://rsbweb.nih.gov/ij/).

#### 2.6.1 Open field test

The mouse was allowed to move freely within an open field apparatus (43 cm × 43 cm) surrounded by transparent plexiglass walls, for a duration of 15 minutes. Tracking of travel distances and durations within both central and peripheral zones was recorded using the ANY-maze software. The animal’s head was designated as the reference point for a zone entry tracking. The central zone was demarcated as a 20 cm × 20 cm area positioned at the center of the enclosure, in alignment with prior descriptions (32). The overall distance traveled reflects the general locomotor activity, while reduced time spent within the center zone signifies tendencies toward anxiety-like behavior (33). A thorough cleansing procedure using 70% ethanol was performed on the apparatus between successive test animals.

#### 2.6.2 Elevated plus maze

The elevated plus maze test is used for measuring anxiety-like behavior in rodents. The test is based on the natural aversion of mice for open and elevated areas, as well as on their natural spontaneous exploratory behavior in novel environments. The EPM apparatus consists of four arms, each with a length of 10 cm × 50 cm, where two arms are open and two are closed. These arms intersect to form a “cross” shape at an elevation of approximately 70 cm above the ground. The mouse was positioned at the intersection area, facing the open arms of the maze, and allowed to explore the maze for a duration of 5 minutes. The movements of each mouse were meticulously tracked and recorded using the ANY-maze software, enabling measurement of the time spent by the mouse in each of the four arms. The number of entries into the open arms and the time spent in the open arms are used as indices of open/elevated space-induced anxiety in mice. Diminished time spent by a mouse in the open arms is indicative of anxiety-like behavior (34).

#### 2.6.3 Light-dark test

The light and dark test is widely used to measure anxiety-like behavior in mice. The test is based on the natural aversion of mice to brightly illuminated areas and on their spontaneous exploratory behavior in novel environments. The LD apparatus contains two compartments: a lit compartment measuring 27 × 27 × 27 cm and a dark compartment featuring blackened walls and floor with dimensions 27 × 18 × 27 cm. These two compartments were separated by a partition with an small opening (7 × 7 cm), enabling the mouse to travel between the two compartments. Each mouse was granted five minutes to explore the entire apparatus, including both compartments. The total duration spent within the lit area was recorded. The number of entries into the compartments and the duration of time spent in each compartment are recorded and used as indices of bright-space anxiety-like behavior in mice. A reduced duration in time spent within the lit compartment suggests an augmentation in anxiety-like behavior (35).

#### 2.6.4 Tail suspension test

The tail suspension test (TST) was performed to evaluate the depression-like behavior in mice. For this assessment, each mouse was suspended by its tail using adhesive tape and fastened to a hook affixed to a metal bar positioned at a height of 45 cm. The metal bar was situated longitudinally between two metal bases. During a 6-minute test session, the cumulative duration of immobility was recorded and measured. The test was conducted concurrently for two animals placed within separate chambers. An extended period of immobility exhibited by a mouse during the TST serves as an indicator of depression-like behavior (36).

#### 2.6.5 Forced swim test

The Forced swim test (FST) was employed to assess depressive-like behavior in mice. The apparatus consisted of a clear Plexiglas cylinder (40 cm in height, 20 cm in diameter) filled with water to a depth of approximately 30 cm (23-25°C). The water was replaced between each trial to ensure consistency. Each mouse was individually placed into the water-filled cylinder and subjected to 6-minute swim session, with a 2-minute pre-test followed by a 4-minute test session. The entire swim session was recorded from a side view. The duration of immobility, which represents a state of behavioral despair, was the primary measure of interest (37).

#### 2.6.6 Object recognition test

The novel object recognition test is predicated on the robust inclination of rodents to preferentially explore novel objects in an open field arena (38). This test encompassed three distinct stages. The first step was the habituation phase, during which the mouse was given five minutes to freely explore the arena (43 cm × 43 cm). For the second stage, two identical objects (each measuring 5 cm × 5 cm × 10 cm) possessing the same texture were positioned within the apparatus, placed at opposing sides with a 5 cm separation from the walls. The mouse was once again placed in the arena and allowed to explore the familiar objects and their surroundings for 5 minutes. Subsequently, the mouse was returned to its home cages for 10 minutes, during which one of the objects was substituted with a different novel object positioned in the same location. The third stage encompasses placing the mouse within the testing arena, allowing it to freely explore both the familiar and the novel object for a duration of five minutes. Time spent exploring the objects were recorded in each stage. Data extracted from the third stage were presented as novel object preference score, calculated as (time exploring novel object/total time for familiar and novel objects exploration) × 100%. The act of exploring the objects was defined as whisking, sniffing, rearing on or touching the object, and approaching the object within a 3 cm proximity.

#### 2.6.7 Social preference test

The social interaction test was conducted within a transparent plastic arena measuring 70 cm × 25 cm × 38 cm, divided into three compartments using two liftable doors. Two cylindrical pencil cups constructed from wire mesh, one containing an adult unfamiliar mouse and the other empty, were placed upside down within the two opposite side chambers (each measuring 28 cm × 25 cm × 38 cm). This arrangement facilitated olfactory interaction while minimized tactile interaction. In the test protocol, each object mouse was initially placed in the empty apparatus and allowed to freely explore the entire apparatus for 5 minutes. Subsequently, an empty cup was placed in one side compartment and a similar cup housing an age- and gender-matched social target mouse in the opposite side compartment and the mouse was allowed to access all the compartments for 10 minutes. Following this session, a novel age- and gender-matched mouse was placed under the empty cup, enabling the object mouse to engage for an additional 10 minutes either with the familiar caged mouse or a novel caged mouse situated in the opposite side compartments. The data extracted from the final phase were represented as a preference score calculated as (time interacting with the novel mouse/total time interacting with both familiar and novel mice) × 100%. Interaction of the object mice with familiar/novel mice was defined by actions such as sniffing, rearing on or touching the pencil cup, and approaching the cup within 3 cm (39).

### 2.7 Immunofluorescence

Mice were sacrificed at 17 weeks of age and subjected to extensive cardiac perfusion with cold PBS followed by 4% paraformaldehyde (PFA). Subsequently, the brain and kidney were carefully harvested and fixed in 4% PFA for 24 hours at 4°C. After dehydration, the brain was embedded within the optimal cutting temperature (OCT) compound, and cryosections were prepared in a coronal plane using a cryosection machine (Leica CM3050 S Cryostat, USA). The frozen sections were subjected to fixation with 4% PFA, followed by permeabilization using 0.5% Triton X-100 in PBS. To inhibit non-specific binding, blocking was executed using 10% goat serum. The sections were subsequently incubated with primary antibodies at different dilutions: 1:800 for DCX (Catalog# 4604S, Cell Signaling Technology), 1:1000 for MeCP2 (Catalog# ab50005, Abcam), 1:200 for glial fibrillary acidic protein (GFAP) (Catalog# 12389S, Cell Signaling Technology), 1:500 for ionized calcium-binding adaptor molecule 1 (Iba1) (Catalog# ab178846, Abcam), 1:200 for neuronal nuclear protein (NeuN) (Catalog# 24307S, Cell Signaling Technology), 1:1000 for CD31 (Catalog# ab24590, Abcam), and 1:500 for Albumin (Catalog# ab207327, Abcam). These brain sections were then incubated with secondary antibodies. For the staining of immune cell infiltration, FITC anti-mouse CD3 Antibody (Clone 17A2, Catalog# 100204, Biolegend) was employed at a dilution of 1:100, and FITC anti-mouse CD19 Antibody (Clone 6D5, Catalog# 115506, Biolegend) at the same dilution. For the IgG deposition test, the frozen sections were directly incubated with goat anti-mouse IgG antibody after the blocking procedure. To counteract fading and label the nuclei, Prolong Gold Antifade Reagent with DAPI (Catalog# 8961s, Cell Signaling Technology) was used. Images were captured using an IX-81 inverted microscope (Olympus) and processed through cellSens Dimension software (Olympus). The acquired results were then subjected to analysis using ImageJ software, including the measurement of dendritic length of the DCX+ newborn neurons, the thickness of the granule cell layer (GCL) of the hippocampus region, and the soma area of the Iba-1 positive macrophages.

### 2.8 TUNEL staining

Terminal deoxynucleotidyl transferase dUTP nick end labeling (TUNEL) staining was conducted utilizing an *in situ* fluorescein-labeled cell death detection kit (Catalog# 11684795910, Roche). Tissue sections were fixed with 4% PFA for 20 minutes, followed by a 30-minute PBS wash. Subsequently, the slides were incubated with permeabilization buffer and then exposed to the TUNEL reaction mixture for labeling. Following the staining procedure, the slides were examined under IX-81 inverted microscope (Olympus), and the captured images were processed using cellSens Dimension software (Olympus).

### 2.9 Western blotting

Whole brain tissues were lysed with protein extraction buffer containing 2% SDS, 25 mM DTT, 100 mM Tris at pH 6.8, and 1x protease/phosphatase inhibitor cocktail (Catalog# 5872S, Cell Signaling Technology). Proteins were run on SDS-PAGE gels with a loading of 40 μg per lane. Proteins were then transferred onto PVDF membranes using the semi-dry transfer method in a transfer buffer consisting of 190 mM Glycine, 25 mM Tris, 0.1% SDS, and 20% methanol. Subsequently, membranes were blocked with 5% non-fat dry milk (Nestle) in TBST buffer containing 20 mM Tris-Cl at pH 7.5, 150 mM NaCl, and 0.1% Tween 20. This was followed by an overnight incubation of primary antibodies at an optimized dilution; refer to **Supplementary Table 2** for more details. The secondary antibodies were diluted at 1:1000 for one-hour incubation at room temperature. Membranes were then developed using a Clarity Western ECL Substrate kit (Catalog# 1705061, Bio-Rad), and a Gel Doc™ XR+ System (Bio-Rad) was used to acquire chemiluminescent western blotting images. Band intensities were quantified through ImageJ software and subsequently normalized against loading control levels of β-actin or GAPDH.

### 2.10 Statistical analysis

The sample size for mice in each test was determined through power analysis. For the analysis of the antigen array test, all data were normalized with the positive control within each array block, followed by logarithmic transformation using base 10. This facilitated the generation of a heatmap that showcased antibody clusters exhibiting similar expression patterns. For the heatmap, proteins were categorized as significantly elevated if their associated P value was below 0.05. Data from each group were imported into R studio for clustering analysis and heatmap construction. The clustering was executed in an unsupervised manner based on Euclidean distance. Moreover, the nonparametric Spearman’s method was used for the correlation analysis between each autoantibody and the anti-dsDNA level. Regarding all other tests, multiple comparisons were performed using one-way analysis of variance (ANOVA), and Tukey’s test was implemented to adjust for confidence intervals and significance. The presentation of aggregate data took the form of means ± SEM. GraphPad Prism software version 9 was used for statistical analysis. A significance threshold of less than 0.05 was adopted for all tests.

## 3 Results

### 3.1 Upregulation of MeCP2 protein in B6.*Mecp2^Tg1^* and spontaneous murine lupus MRL/*lpr* mice

**Figure 1:**
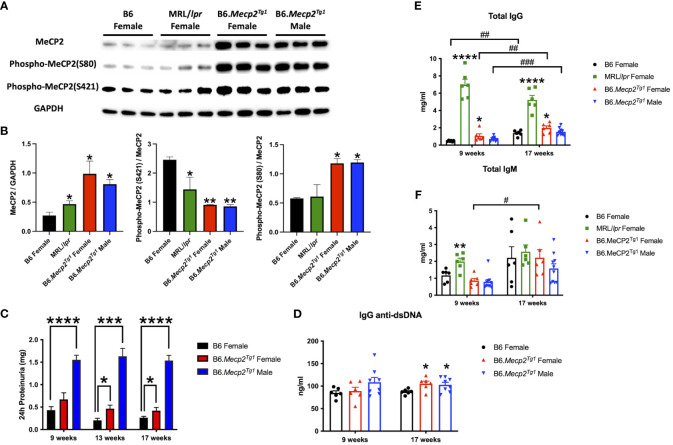
MeCP2 overexpression mice exhibit elevated proteinuria and immunoglobulins. **(A, B)** Expression of MeCP2 and its two phosphorylated forms within the brain of all mice groups, along with the normalization analysis. **(C)** Quantification of 24-hour proteinuria across different age intervals for the four mice groups. **(D-F)** Assessment of IgG anti-dsDNA antibody, total IgG, and IgM levels at both the 9-week-old and 17-week-old for the mice. n = 5-12 per group. *, *P* < 0.05, **, *P* < 0.01, ***, *P* < 0.001, ****, *P* < 0.0001. #, *P* < 0.05, ##, *P* < 0.01, ###, *P* < 0.001. * means changes between mouse groups, and # means changes within each mouse group.

To determine MeCP2 protein expression in murine lupus, we performed a western blot analysis using the brain tissue lysates from the spontaneous lupus mouse model MRL/*lpr*. The protein expression of MeCP2 and its two phosphorylated forms within the brain of MRL/*lpr* and *Mecp2* transgenic mice was examined, as shown in **Figures 1A, B**. The overall MeCP2 protein expression level was significantly increased in MRL/*lpr* mice, as compared to the wildtype B6 control (**Figures 1A, B**). This result strongly suggests that MeCP2 is indeed upregulated in murine lupus. Interestingly, both female and male B6.*Mecp2^Tg1^* mice displayed a significant increase in pan-MeCP2 and phospho-MeCP2 at S80 while displaying a reduction in phospho-MeCP2 levels at S421 within the brain compared to B6 (**Figures 1A, B**).

### 3.2 Autoimmune phenotypes in B6.*Mecp2^Tg1^* mice

The litters from the B6.*Mecp2^Tg1^* breeding pair were genotyped, and only mice carrying the transgene were used for further experiments (**Supplementary Figure 1A**). As shown in **Supplementary Figure 1B**, the spleen weights of both genders of the transgenic mice were similar when compared to B6 controls. 24-hour proteinuria was measured at different time points in the transgenic mice. Intriguingly, male B6.*Mecp2^Tg1^* mice displayed elevated proteinuria levels as early as 9-week-old, while female B6.*Mecp2^Tg1^* mice exhibited a notable increase in proteinuria starting at 13 weeks of age (**Figure 1C**). The 24-hour proteinuria data for all mice groups at 17 weeks of age, including lupus-prone control MRL/*lpr* mice, was summarized in **Table 1**.

**Table 1:** 24h Proteinuria at 17 weeks (mg).

Strain	Gender	N	Mean	SEM	P value compared to B6 (two-tailed)
B6	F	11	0.2643	0.02931	
MRL/*lpr*	F	6	3.442	1.182	0.002
B6.*Mecp2^Tg1^*	F	7	0.4214	0.07174	0.0331
B6.*Mecp2^Tg1^*	M	12	1.49	0.1165	<0.0001

Both genders of the transgenic mice exhibited enhanced levels of IgG anti-dsDNA in comparison to B6 controls at 17 weeks of age, aligning with the profile observed in MRL/*lpr* mice (**Figure 1D**, **Table 2**). Remarkably, the total IgG level was notably upregulated in early-aged female B6.*Mecp2^Tg1^* mice compared to B6, and this elevation persisted through the 17 weeks of age point (**Figure 1E**). The male transgenic mice displayed elevated IgG level over time, although not significantly compared to B6. Furthermore, **Figure 1F** indicates a considerable elevation in IgM levels over time in female B6.*Mecp2^Tg1^*. Collectively, these findings suggest that similar to the MRL/*lpr*, both male and female B6.*Mecp2^Tg1^* mice display lupus-like phenotypes, including elevated proteinuria, augmented anti-dsDNA antibodies, and increased total IgG/IgM levels.

**Table 2:** IgG anti-dsDNA level at 17 weeks (ng/ml).

Strain	Gender	N	Mean	SEM	P value compared to B6 (two-tailed)
B6	F	6	87.54	2.408	
MRL/*lpr*	F	6	6148	1219	0.0006
B6.*Mecp2^Tg1^*	F	6	105.4	5.802	0.0177
B6.*Mecp2^Tg1^*	M	8	103	5.423	0.039

Given the notable increase of 24-hour proteinuria observed in the *Mecp2* transgenic mice, we performed immunofluorescence to detect potential pathological alterations in the B6.*Mecp2^Tg1^* mice kidneys. As shown in **Supplementary Figure 1C**, an enhanced deposition of IgG was observed in the kidney glomerulus area in female B6.*Mecp2^Tg1^* mice when compared to B6 controls. Furthermore, pronounced macrophage infiltration into the kidneys of female *Mecp2* transgenic mice was evident, in contrast to B6 controls (**Supplementary Figure 1D, E**). Collectively, aggregated IgG deposition, increased macrophage infiltration, and elevated proteinuria confirmed the progressive renal impairment observed in *Mecp2* transgenic mice, akin to the phenotypes associated with lupus nephritis.

### 3.3 Autoantibody profiling in B6.*Mecp2^Tg1^* mice

**Figure 2:**
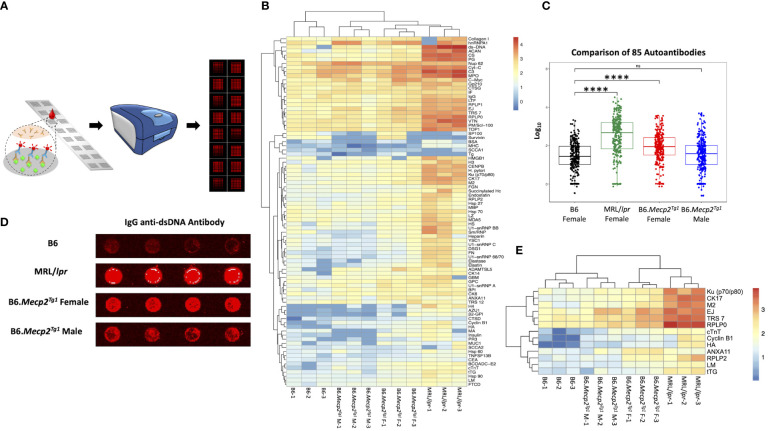
Comprehensive autoantibody profiling across all mice groups at 17-week-old. **(A)** A schematic depiction of the workflow of the autoantibody array, utilizing fluorescence labeling for detection. **(B)** Unsupervised HeatMap cluster analysis for the autoantibody levels in all mice groups, effectively demonstrating distinct clustering patterns. **(C)** Overall comparison of the total 85 autoantibody IgG levels. **(D)** An example of the IgG anti-dsDNA antibody signal presented on the array slides, wherein antigens were printed in quadruplicate. **(E)** Representation of remarkably elevated antibodies in both MRL/lpr and female transgenic mice when compared to B6. n = 3 per group at 17-week-old. ****, *P* < 0.0001. “ns” means no significant change.

Next, we employed an in-house autoantigen array harboring 85 antigens to comprehensively screen for autoantibodies across all mouse groups. As shown in **Figure 2A**, each autoantigen array chip is subdivided into 2 x 8 subarrays, enabling the concurrent assessment of 16 samples per assay. Serum samples obtained from mice in each group were applied to the array, with Cy5 labeled anti-mouse IgG used to recognize the antigen-antibody complexes on the array surface. Subsequently, an unsupervised heatmap cluster method was applied to interpret the outcomes of the antigen array test. The resulting clustering of all three mouse strains accurately and distinctly identified each group, revealing a hierarchical order: MRL/lpr > B6.*Mecp2^Tg1^* > B6 (**Figures 2B, C**). Comprehensive statistical analyses comparing the groups are shown in **Supplementary Table 3**. A representative array image of IgG anti-dsDNA antibody is shown in **Figure 2D**. Notably, female B6.MeCP2^Tg1^ mice exhibited higher autoantibody levels compared to B6 (**Figures 2B-D**). Interestingly, 59 out of 85 autoantibodies displayed notable correlations with anti-dsDNA antibody levels within each mouse group (**Supplementary Table 3**). Among all autoantibodies tested, 13 of them exhibited strikingly elevation in both the MRL/*lpr* and female transgenic mice compared to B6, including the anti-ribosomal P0 (RPLP0), a key CNS autoantibody in NPSLE (**Figure 2E**). Other significantly elevated autoantibodies including Ku (p70/p80), CK17, M2, EJ, TRS7, cTNT, Cyclin B1, HA, ANXA11, RPLP2, LM and tTG. These findings collectively underscore that B6.*Mecp2^Tg1^* mice, particularly the females, manifest a remarkable upsurge of autoantibodies, similar to a spontaneous murine lupus strain MRL/*lpr*.

### 3.4 Immune cell subsets and activation status in B6.*Mecp2^Tg1^* mice

**Figure 3:**
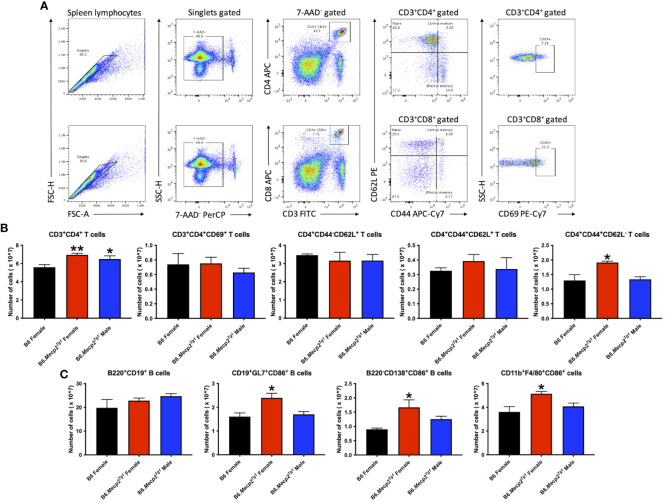
Cell numbers are remarkably changed in CD3^+^CD4^+^ T cell subsets and activated B cell subsets in the splenocytes from female B6.*Mecp2^Tg1^* mice compared to B6. **(A)** Gating strategy of T cell subsets. **(B)** Cell number changes of CD3^+^CD4^+^ and its activation form, as well as the changes of its subsets CD4^+^CD44^-^CD62L^+^ naïve T cells, CD4^+^CD44^+^CD62L^+^ central memory T cells, and CD4^+^CD44^+^CD62L^-^ effector memory T cells. **(C)** Cell number changes of B220^+^CD19^+^ total B cells, CD86^+^ labeled activated CD19^+^GL7^+^ germinal center cells, B220^-^CD138^+^ plasma B cells, and CD11b^+^F4/80^+^ macrophages. n = 3 per group at 17-week-old. *, *P* < 0.05, **, *P* < 0.01.

Flow cytometry was implemented to characterize immune cell phenotypes in splenocytes from the transgenic mice and B6 controls, elucidating alterations in T cells, B cells, myeloid cells, and their respective subsets in the B6.*Mecp2^Tg1^* mice. **Figure 3A** shows the gating strategy for CD3^+^ T cells and their subsets, including CD3^+^CD4^+^ or CD3^+^CD8^+^ T cells, along with their activated CD69^+^ forms. Notably, the total count of CD3^+^CD4^+^ T cells was significantly upregulated in B6.*Mecp2^Tg1^* mice compared to B6 controls while the CD3^+^CD4^+^CD69^+^ activated T cells did not show any substantial changes. No remarkable differences were observed between transgenic mice and B6 controls in CD3^+^CD4^+^CD44^-^CD62L^+^ naïve T cells or CD3^+^CD4^+^CD44^+^CD62L^+^ central memory T cells. Conversely, the population of CD3^+^CD4^+^CD44^+^CD62L^-^ effector memory T cells was notably increased, particularly in female B6.*Mecp2^Tg1^* mice (**Figure 3B**). For CD3^+^CD8^+^ T cells, neither the overall count nor any specific cell subsets showed significant changes in the transgenic mice (**Supplementary Figure 2A**). In addition, the number of B220^+^CD19^+^ total B cells, CD21^int^CD23^high^ follicular B cells, CD21^high^CD23^low^ marginal zone B cells, CD19^+^GL7^+^ germinal center B cells, and B220^-^CD138^+^ plasma B cells exhibited no meaningful changes in the transgenic mice when compared to B6 (**Figure 3C**; **Supplementary Figure 2B**). Interestingly, in the female B6.*Mecp2^Tg1^* mice, there was a marked increase in the population of CD86^+^ labeled activated germinal center B cells (CD19^+^GL7^+^CD86^+^) and CD86^+^ labeled plasma B cells (B220^-^CD138^+^CD86^+^) when compared to B6 controls (**Figure 3C**). Furthermore, the quantities of CD11b^+^F4/80^+^ macrophages, CD11c^-^CD11b^+^ cells, and CD11c^+^CD11b^+^ dendritic cells remained relatively unchanged in the transgenic mice; however, the female transgenic mice exhibited a notable increase in the count of CD86^+^ labeled activated CD11b^+^F4/80^+^CD86^+^ macrophages compared to B6 controls (**Supplementary Figure 3B**; **Figure 3C**). Additionally, the CD4^+^CD25^+^ regulatory T cells did not display any significant differences between transgenic and B6 control mice (**Supplementary Figure 3A**), as well as CD93^+^CD23^-^ T1 B and CD93^+^CD23^+^ T2 B cells (**Supplementary Figure 3A**).

In summary, the increase of splenic effector memory T cells, and the surge of activated germinal center B cells, plasma cells, and macrophages collectively indicated an expanded immune cell population and an activated immune system in the B6.*Mecp2^Tg1^* mice.

### 3.5 Anxiety-like behavior and locomotor dysfunction in B6.*Mecp2^Tg1^* mice

**Figure 4:**
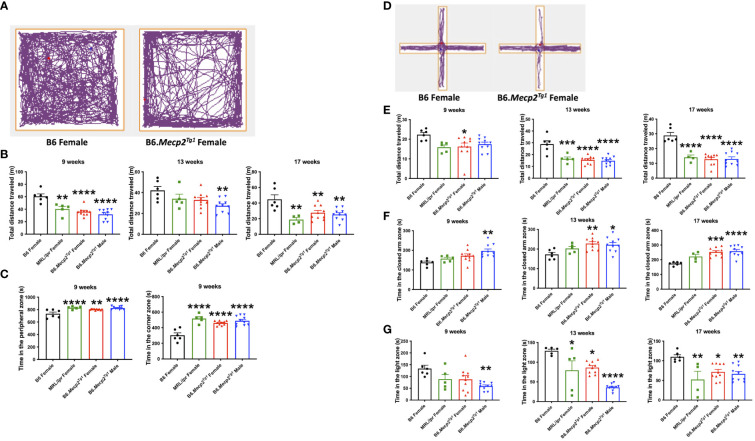
Behavioral assessments indicate decreased locomotor activity and anxiety-like behavior in *Mecp2* transgenic mice. **(A)** The representative movement tracking for B6 and female transgenic mice in the open field test. **(B)** The total distance that mice traveled in the entire open field arena at different time points. **(C)** Time in the peripheral zone and corner zones was spent by the mice at 9-week-old. **(D)** Representative movement tracking for B6 and female transgenic mice in the elevated plus maze test. The orange line demarcates different zones. **(E)** Total distance traveled by mouse in the elevated plus maze at different time points. **(F)** Time spent by mice in the closed arms at different time points. **(G)** Time spent by mice on the light side in the light-dark test at different time points. n = 6 for B6 and MRL/*lpr* mice; n=10 for both transgenic groups. *, *P* < 0.05, **, *P* < 0.01, ***, *P* < 0.001, ****, *P* < 0.0001.

Next, we conducted an open field test to assess anxiety-like behavior and locomotor activity in the transgenic mice. The open field area was divided into three distinct zones: the central, peripheral, and corner zones. The mouse movement was traced and presented (**Figure 4A**). The results distinctly indicate a remarkable decline in the overall distance traveled by the transgenic mice compared to the B6 controls. Similar reduction in locomotion was observed in spontaneous lupus mouse strain MRL/*lpr* as early as 9 weeks of age (**Figure 4B**). Moreover, the prolonged duration of the transgenic mice spent in both the corner and peripheral zones of the open field arena indicated an anxiety-like syndrome and diminished locomotor activity (**Figure 4C**).

The elevated plus maze was used as an additional tool for the assessment of anxiety-like behavior. This maze consists of three zones: the central zone, open arm zone, and closed arm zone. Typical tracing of the mouse movement was shown in **Figure 4D**. A notable decrease in total distance traveled was observed in female transgenic mice as early as 9 weeks of age, with similar changes emerging in the MRL/*lpr* and male transgenic mice by 13 weeks of age (**Figure 4E**). Additionally, male transgenic mice spent significantly more time in the closed arm zone compared to B6 at an early age, whereas a striking increase of time spent in the closed arms was observed at 13 weeks of age for both genders of the transgenic mice and persisted till 17 weeks of age (**Figure 4F**).

Another test used to interrogate anxiety-like behavior was the light-dark box test. This apparatus comprises two chambers: a transparent light side except for the partition between two sides, and a concealed dark side where the mouse is invisible when inside. A distinct reduction in the time that male transgenic mice spent on the light side was observed at 9 weeks of age. MRL/*lpr* and female transgenic mice displayed a notable reduction in time spent on the light side starting at 13 weeks of age (**Figure 4G**). Collectively, these three tests revealed compromised locomotor activity and the emergence of anxiety-like behavior in the transgenic mice at an early age compared to B6 controls.

### 3.6 Depression-like behavior in B6.*Mecp2^Tg1^* mice

**Figure 5:**
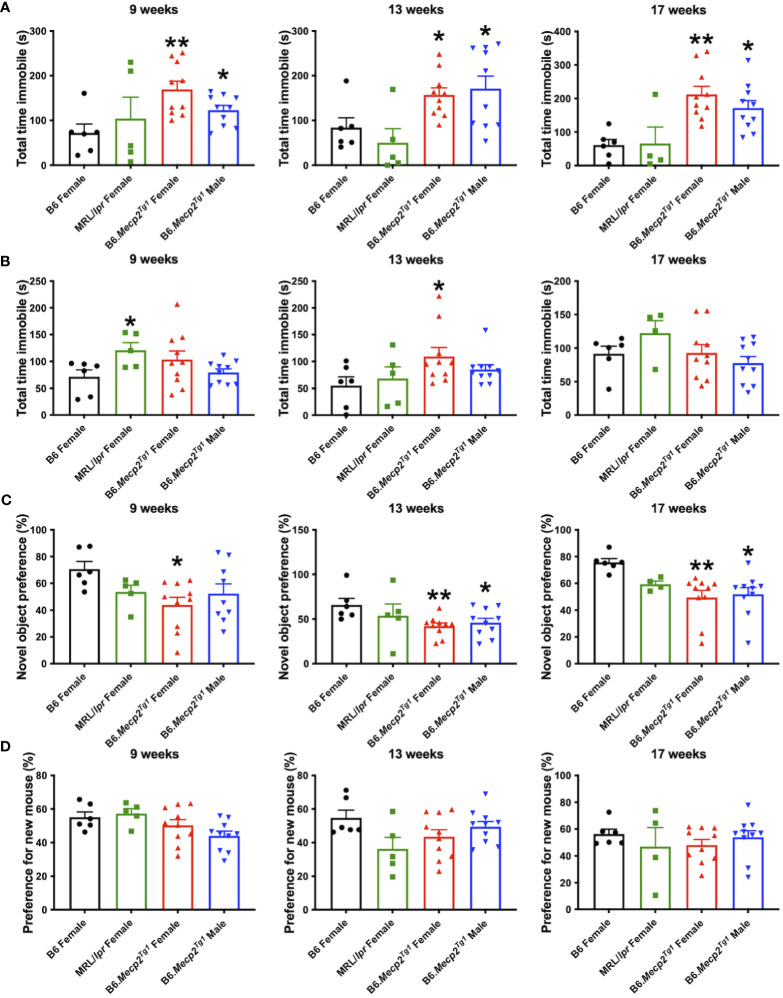
Depression-like behavior and impaired cognitive ability in B6.*Mecp2^Tg1^* mice. **(A, B)** Immobile time was detected in the tail suspension test and forced swim test at different time points, respectively. **(C)** Novel object preference was determined in each group of mice at different time points. **(D)** Preference for the novel social interaction partner mouse was measured at different time points. n = 6 for B6 and MRL/*lpr* mice; n=10 for both transgenic groups. *, *P* < 0.05, **, *P* < 0.01.

Durations of immobility were observed and recorded during the tail suspension test and forced swim test. Intriguingly, both male and female B6.*Mecp2^Tg1^* mice displayed prolonged immobile time than B6 controls at 9 weeks of age in the tail suspension test. This phenotype persisted until 17 weeks of age (**Figure 5A**). Moreover, in comparison with B6 controls, the immobile time of female B6.*Mecp2^Tg1^* mice were remarkably increased at 13 weeks of age in the forced swim test (**Figure 5B**). Results from these two tests collectively indicate the presence of depression-like behavior in B6.*Mecp2^Tg1^* mice.

### 3.7 Compromised cognitive and memory function in B6.*Mecp2^Tg1^* mice

To study the potential influence of MeCP2 overexpression on recognition and memory function of the mice, we conducted the object recognition test to assess their short-term memory capabilities. We found female B6.*Mecp2^Tg1^* mice presented significantly reduced preference for the novel object compared to B6 controls, starting at 9-week-old. However, the male B6.*Mecp2^Tg1^* mice displayed a slight delay in the onset of reduced novel object preference (**Figure 5C**). Subsequently, the social abilities of these mice were also evaluated through the social interaction test. Similar to the object recognition test, preference for the novel mouse was used to assess the social ability across all mice groups. Interestingly, neither MRL/*lpr* mice nor the *Mecp2* transgenic mice exhibited abnormality in novel mouse preference, indicating intact social abilities in these mice groups (**Figure 5D**).

Through all the behavior tests above, we found that the B6.*Mecp2^Tg1^* mice exhibited an early onset of multiple abnormal behaviors, including reduced locomotor activity, the onset of anxiety-like behavior and depression-like behavior, as well as compromised recognition and short-term memory abilities.

### 3.8 Impaired neurogenesis in the hippocampus region of B6.*Mecp2^Tg1^* mice

**Figure 6:**
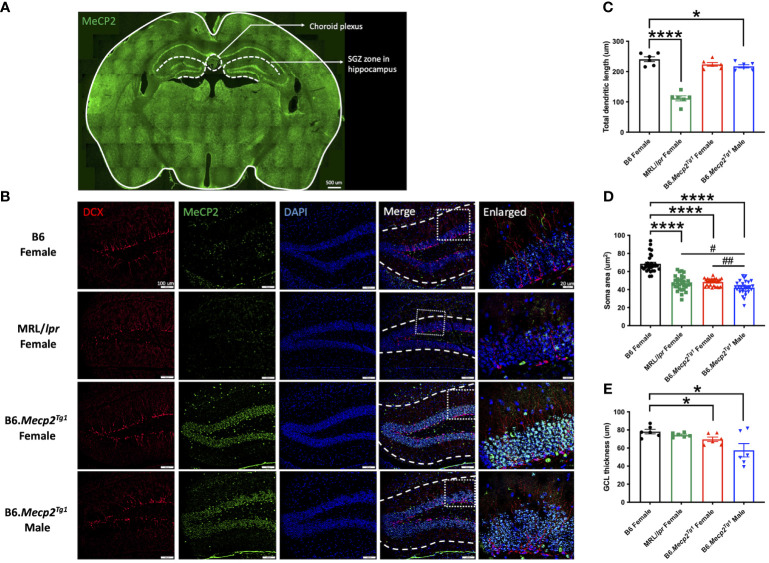
Impact of MeCP2 overexpression on neurogenesis in the hippocampus. **(A)** MeCP2 expression in a whole-brain section of a female B6.*Mecp2^Tg1^* mouse. Green, MeCP2. Dashed lines demarcate distinct functional brain regions. Scale bar: 500 μm. **(B)** Assessment of neurogenesis within the SGZ of the hippocampus region. Red, doublecortin (DCX) labeled newborn neurons; green, MeCP2; blue, DAPI labeled nuclei; fourth column, merged images from the preceding three columns. White dashed lines in the fourth column delineate the extent of dendrite length in newborn neurons. Scale bar: 100 μm. The fifth column displays an enlarged view of the area marked by the dashed rectangular in the previous column. Scale bar: 20 μm. **(C-E)** Analysis of total dendritic length, soma area of newborn neurons, and the thickness of granule cell layer (GCL). Three mice per group were studied at 17-week-old, and both sides of the hippocampus were analyzed. *, *P* < 0.05, ****, *P* < 0.0001; #, *P* < 0.05, ##, *P* < 0.01. * means a significant difference compared to B6, and # means notable difference compared to MRL/*lpr* mice.

To comprehend the neuropathology underlying the observed behavioral changes in the *Mecp2* transgenic mice, we delved into potential pathological changes across various brain regions using immunofluorescence. Specifically, we focused on the subgranular zone (SGZ) within the mouse hippocampus and the choroid plexus region (**Figure 6A**). The MeCP2^+^/DAPI^+^ cells (cyan colored) exhibited predominant expression exclusively within the transgenic mice, with notable localization of MeCP2 primarily within the granule cell layer (GCL) of the hippocampus region (**Figure 6B**). Compared to B6, the total dendritic length of newborn neurons was notably diminished in the male *Mecp2* transgenic mice, accompanied by a reduction in the soma area in both genders of the *Mecp2* transgenic mice. These changes were mirrored in the MRL/*lpr* mice as well (**Figures 6B–D**). More importantly, both female and male transgenic mice displayed a more pronounced decline of soma area when compared to MRL/*lpr* (**Figure 6D**). Furthermore, the thickness of the granule cell layer (GCL) was also remarkably decreased in the *Mecp2* transgenic mice (**Figure 6E**). Collectively, these findings provide compelling evidence of compromised neurogenesis in the *Mecp2* transgenic mice.

Next, we investigated the specific cell type responsible for MeCP2 expression within the brain. Double immunofluorescence staining was performed to observe the co-localization of MeCP2 with major neural cell markers in the SGZ of a female transgenic mouse. The utilized neural cell markers encompassed glial fibrillary acidic protein (GFAP) for astrocytes, ionized calcium-binding adaptor molecule 1 (Iba1) for microglia, doublecortin (DCX) for newborn neurons, and neuronal nuclear protein (NeuN) for mature neurons. The findings demonstrated pronounced MeCP2 expression in NeuN-positive mature neurons, while no MeCP2 expression was observed in other cell types (**Supplementary Figure 4**).

### 3.9 Neuroinflammation in the brain of B6.*Mecp2^Tg1^* mice

**Figure 7:**
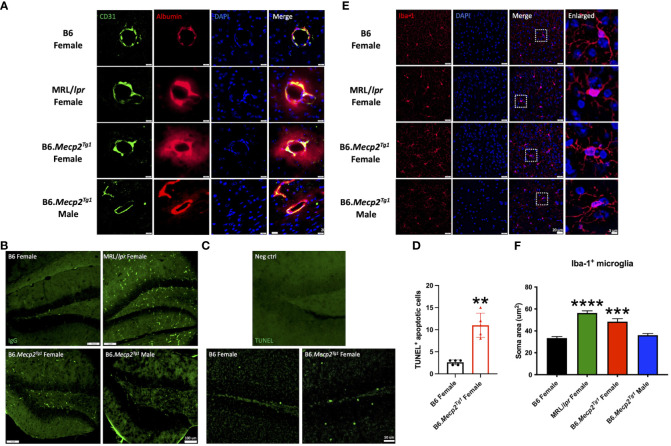
Neuroinflammation observed in female B6.*Mecp2^Tg1^* mice. **(A)** Dual staining of CD31 labeled endothelial cells and albumin. Green, CD31 labeled endothelial cells; red, albumin; blue, DAPI labeled nuclei; the fourth image displays the merged view of the preceding images. Scale bar: 20 μm. **(B)** Immunofluorescence staining for total IgG in the hippocampus of all mice groups. Green, IgG. Scale bar: 100 μm. **(C)** Identification of apoptosis through TUNEL labeling in the hippocampus region of both B6 and female transgenic mice. The brain section from the female transgenic mouse incubated with label solution instead of the TUNEL reaction mixture was used as a negative control. Green, TUNEL labeled apoptotic cells. Scale bar: 50 μm. **(D)** Statistical analysis of TUNEL^+^ apoptotic cells in the hippocampus region. n = 5 per group at 17 weeks of age. **, *P* < 0.01. **(E)** Immunofluorescence staining of the Iba-1 labeled microglia. Red, Iba-1 labeled microglia; blue, DAPI labeled nuclei; The third image combines the views of the preceding images. Scale bar: 20 μm. The fourth image provides an enlarged view of the regions enclosed by dashed rectangles in the previous image. Scale bar: 5 μm. **(F)** Soma area analysis of Iba-1 labeled microglia. Three mice per group were studied, with a total of 30 microglia cells analyzed in each mouse group. ***, *P* < 0.001, ****, *P* < 0.0001.

Prior results showed that the MeCP2 overexpression might impact the neurogenesis in the transgenic mice. However, the relationship was not direct, as the protein was found to be exclusively expressed in mature neurons. Thus, we investigated another potential contributing factor to neurogenesis, neuroinflammation, in the mouse brain. Through co-labeling of CD31-marked endothelial cells and albumin, we observed expanded albumin diffusion across the blood vessels in the *Mecp2* transgenic mice, similar to the pattern observed in MRL/*lpr*. This finding indicated a disruption in the integrity of the blood-brain barrier (BBB) in the transgenic mice (**Figure 7A**). Meanwhile, increased IgG deposition and TUNEL-labeled apoptotic cells were also detected in the hippocampus of female transgenic mice (**Figures 7B, C**). The number of TUNEL^+^ apoptotic cells was significantly higher in the female transgenic mice compared to the B6 controls (**Figure 7D**). These results strongly imply that the inflammatory response associated with BBB disruption, accumulated IgG deposition, and augmented apoptosis may contribute to the compromised neurogenesis in B6.*Mecp2^Tg1^* mice.

Furthermore, the microglia in both MRL/*lpr* and female B6.*Mecp2^Tg1^* mice displayed an activated morphology, transitioning from a highly ramified structure to an amoeboid cell shape (**Figure 7E**). Our statistical analysis further confirmed an enlarged microglia soma area in female B6.*Mecp2^Tg1^* and MRL/*lpr* mice compared to B6 (**Figure 7F**). Collectively, these findings provide additional evidence supporting the presence of neuroinflammation in the hippocampus region of *Mecp2* transgenic mice, particularly in females.

### 3.10 Immune cell infiltration in the choroid plexus of B6.*Mecp2^Tg1^* mice

The choroid plexus (CP), a highly vascularized capillary plexus in the brain surrounded by a cuboidal epithelial bilayer, produces most of the cerebrospinal fluid (CSF) of the central nervous system. Particularly, it has been shown that the CP hosts a population of CD4^+^ T cell population and experiences IgG deposition during CNS infection (40). We examined the inflammation in the CP area using immunofluorescence, including the immune cell infiltration and the IgG deposition. Our observations indicated a significant increase in the accumulation of CD3^+^ T cells and CD19^+^ B cells in female B6.*Mecp2^Tg1^* mice, similar results were observed in MRL/*lpr* mice (**Supplementary Figures 5A, B**). This trend was also reflected in the IgG deposition within these mice groups (**Supplementary Figure 5C**). Interestingly, although the hippocampus is close to the CP in the anatomy, no detectable T or B cells were observed within its confines (data not shown). The immune cells enrichment and IgG deposition in the CP area, consistent with the IgG deposition and microglia activation in the hippocampus region, collectively point towards a severe CNS inflammation in the female B6.*Mecp2^Tg1^* mice.

### 3.11 Potential mediation of key signaling molecules in lupus pathogenesis by MeCP2

**Figure 8:**
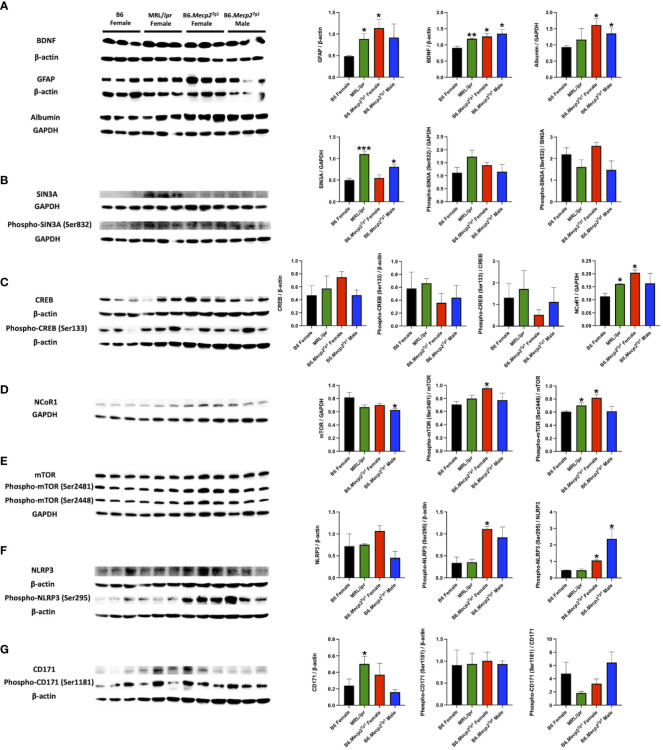
Brain signaling pathways impacted by MeCP2 overexpression. **(A)** Brain albumin, BDNF, and GFAP expression and normalization in the four mice groups. Protein extraction was performed on whole brain tissue. **(B-D)** Expression and statistical analysis for MeCP2 related proteins expression in the brain. **(E-G)** Expression and normalization of signaling molecules implicated in lupus and their phosphorylation forms. n = 3 per group at 17-week-old. *, *P* < 0.05, **, *P* < 0.01.

MeCP2 operates as a multifaceted regulator of gene expression and chromatin organization (17, 41). Notably, proteins implicated in CNS disorders, such as brain-derived neurotrophic factor (BDNF), GFAP, and albumin, displayed elevated levels in the brains of both B6.*Mecp2^Tg1^* and MRL/*lpr* mice compared to B6 controls (**Figure 8A**). Moreover, crucial transcriptional regulators related to lupus such as HDACs/Sin3A, cAMP-responsive element-binding protein (CREB), and nuclear receptor corepressor 1 (NCoR1) were upregulated in both B6.*Mecp2^Tg1^* and MRL/*lpr* when compared to B6 controls (**Figure 8B–D**). The two phosphorylation forms of mTOR (Ser2481 and Ser2448) were also upregulated in the transgenic mice (**Figure 8E**). Proteins from previous studies that associated with lupus such as NLRP3 (**Figure 8F**), CD171 (**Figure 8G**), and others (**Supplementary Figures 6A, B**) were also examined. Remarkably, both phospho-NLRP3 (Ser295) and CD171 displayed elevated levels in B6.*Mecp2^Tg1^* and MRL/*lpr* mice compared to B6 controls. A summary of the examined signaling molecules in the transgenic mice is listed in **Supplementary Figure 6C**, only PPP2R2B exhibited significant decrease in the male transgenic mice compared to B6 (**Supplementary Figure 6B**). These results imply that MeCP2 may potentially mediate key signaling molecules implicated in the pathogenesis of lupus.

## 4 Discussion

Several spontaneous murine models of SLE have been used in NPSLE studies, including MRL/*lpr*, NZB/NZW F1, BXSB, and B6.*Sle1.Sle3*, B6.*lpr*, *etc*. However, none of these models are ideal for recapitulating human NPSLE, particularly with regard to the robustness of neuropsychiatric phenotypes and behavioral deficits (1, 42). Hence, there is an urgent need to identify a novel NPSLE mouse model to aid in uncovering the disease mechanism and discovering new drug targets.

MeCP2 plays a pivotal role in the epigenetic regulation of methylation-sensitive genes and is implicated in the pathogenesis of neurological diseases such as RTT (14–17). Studies from various clinical cohorts suggest that *Mecp2* duplication syndrome may explain the X-linked mental retardation, accompanied by features such as dysmorphic characteristics, developmental delays, minimal speech, recurrent infections, spasticity, autism traits, epilepsy, and possible developmental regression. Males affected may display a range of variable phenotypes, with nearly 40% reported to have died at an early age (43). In females with *Mecp2* duplication, disease severity exhibited significant skewing in X-chromosome inactivation, with the mother showing milder phenotypes compared to her twin daughters (44). Interestingly, genetic polymorphisms in *Mecp2* have also been identified in SLE patients (23, 24). Despite these connections, the expression, phosphorylation, and downstream signaling of MeCP2 in the context of SLE have yet to be explored. In this study, we present the first evidence of significant MeCP2 overexpression in the brains of a spontaneous lupus model, MRL/*lpr*. This result prompted us to further investigate the functional connections between MeCP2 overexpression and the underlying mechanisms of NPSLE pathogenesis. *Mecp2* duplication on the FVB/N mouse background exhibited elevated serum antinuclear antibody (ANA) (26). However, the systemic immune dysregulation in these mice remains unexplored, including specific immune cell activation, immunoglobulin deposition in the kidney, or proteinuria. Furthermore, the presence of premature retinal degeneration that develops on the FVB/N background complicates the accurate assessment of disease phenotypes associated with MeCP2 overexpression (45). In the present study, we utilized a *Mecp2* transgenic mouse model on the C57BL/6 background, enabling us to accurately investigate these lupus-like phenotypes. It is of interest to note that female B6.*Mecp2^Tg1^* mice exhibited more pronounced elevations in total serum IgG levels, macrophage infiltration into the kidney, and IgG deposition within the kidney, in comparison to B6, while the male transgenic mice remained unchanged. This divergence aligns with the gender bias as commonly seen in human lupus and other lupus mouse models (46). Moreover, it is noteworthy that we were able to perform autoantibody profiling on the *Mecp2* transgenic mice using an in-house autoantibody microarray platform (29–31). Our investigation unveiled that 13 autoantibodies were significantly elevated in both MRL/*lpr* and female B6.*Mecp2^Tg1^* mice when compared to B6 controls. This indicates that B6.*Mecp2^Tg1^* mice exhibit a broad production of lupus-related autoantibodies, thereby resulting in lupus-like phenotype. Furthermore, CD3^+^CD4^+^ T cells were found to be significantly increased in the female B6.*Mecp2^Tg1^* mice, implying their contribution to the enhanced immune responses in the transgenic mice. Germinal center B cells were demonstrated to be actively engaged in antigen recognition and pre-primed for differentiation into antibody-producing plasma cells (47, 48). Our investigation unveiled a substantial elevation in CD86+ activated germinal center B cells and plasma cells, elucidating the mechanisms underlying autoantibody production in the transgenic mice. Meanwhile, we observed an overpopulation of activated CD11b^+^F4/80^+^ macrophages in the spleen, indicating presence of inflammation in the spleen of female transgenic mice. Our results are consistent with prior research that identified F4/80^+^ macrophage infiltration in the red pulp of inflamed spleens (49). Renal CD11b^+^F4/80^+^ population were proved to be located throughout the interstitium, displayed an aberrant activation profile contribute to tissue damage in lupus nephritis by mediating both local inflammation and excessive tissue remodeling (50).

In a previous study, a series of behavioral tests, including the rotarod test, open field test, elevated plus maze test, and social behavior test were conducted, to investigate behavior changes in *Mecp2* transgenic mice on an FVB background. Notably, this study demonstrated that correcting MeCP2 levels using an antisense oligonucleotide approach could effectively reverse the behavioral, molecular, and electrophysiological deficits observed in these MeCP2 duplication mice (51). Our current study validated behavioral changes induced by MeCP2 overexpression in B6.*Mecp2^Tg^* ^1^ mice, including the emergence of anxiety-like behavior and reduced locomotor ability, while rodent social abilities remained unchanged. Additionally, we expanded our assessment with additional behavioral tests, such as the light-dark test, forced swim test, tail suspension test, and novel subject recognition test on B6.*Mecp2^Tg^* ^1^ mice. Through these tests, we further observed increased levels of depression-like behavior, impaired recognition and short-term memory in B6.*Mecp2^Tg1^* mice when compared to B6 controls.

We further examined the impact of MeCP2 overexpression at the micromorphological level. The hippocampus is a pivotal brain region characterized by ongoing neurogenesis that occurs throughout adulthood, significantly contributing to learning, memory (52), and emotional regulation (53, 54). Significant disparities in neurogenesis between *Mecp2* transgenic mice and the B6 controls were observed, including variations in dendritic length and abnormal soma area of DCX^+^ newborn neurons, as well as a reduction in the thickness of the GCL formed by mature neurons. Given the role of adult hippocampal neurogenesis in memory and learning, this disrupted neurogenesis may contribute to the learning-memory and social impairment observed in B6.*Mecp2^Tg1^* mice, along with abnormal MeCP2 expression that affecting neuronal function.

The blood-brain barrier (BBB) is located at the interface between the CNS and the circulatory system and plays a pivotal role in establishing and maintaining the microenvironmental homeostasis of the CNS. Evidence has consistently shown that disruptions in the BBB could promote inflammation by facilitating the migration of immune cells through both the paracellular and transcellular pathways, thereby infiltrating the CNS parenchyma (55). A previous study also demonstrated that lupus-prone strains, including NZB/NZW and a lupus-prone mouse strain dependent on interferon-α receptor 1 (IFNAR), namely 564Igi mice, exhibited an increase in the number of reactive microglia (56). The activation of microglia has been shown to be a critical mediator of neuronal damage in lupus-prone mice (57). Our study unveiled a significant diffusion of intravascular albumin in the transgenic mice, similar to that observed in MRL/*lpr*. Additionally, we noted the presence of IgG deposition in the hippocampus area. These findings suggest that the BBB leakage may induce inflammation in the mouse brain, a notion further validated by the microglia activation. Moreover, in a previous autoimmune-prone mouse model, cytokine B-cell-activating factor (BAFF) transgenic mice, the anxiety behavior observed on the mice was proven to be associated with neuroinflammation, impaired neurogenesis, and hippocampal plasticity (58). Our antigen array shows that the brain-reactive autoantibodies were significantly elevated in the female transgenic mice compared to B6 controls, and this finding is highly correlated with the impairment of spatial memory and cognitive dysfunction.

The choroid plexus (CP) is a densely vascularized structure consisting of endothelial-epithelial convolutions in the ventricular system. Its primary function is the production of cerebrospinal fluid (CSF), and it forms a crucial part of the blood-CSF barrier (59, 60). Prior research has associated T cell infiltration within the CP region with the emergence of depressive-like behavior and impaired cognition in the MRL/*lpr* model (61). Our results further characterize the IgG deposition and immune cell infiltration in the CP region, consistent with previous studies in the hippocampus area.

Meanwhile, the MeCP2 phosphorylation is known to play a role in learning and memory, as evidenced by previous studies (62, 63). S421 phosphorylation of MeCP2 could be selectively induced in the brain in response to physiological stimuli. This phosphorylation at S421 enables MeCP2 to regulate crucial aspects of neuronal function, such as dendritic patterning, spine morphogenesis, and activity-dependent induction of BDNF transcription (64). More importantly, both phosphorylation at S421 and dephosphorylation at S80 of MeCP2 are observed to be triggered by neuronal activity (65). Disabled S80 phosphorylation reduces MeCP2’s association with chromatin, consequently altering the transcription genes that potentially hold significance for neuronal function (66). Interestingly, our observations revealed a remarkable increase in S80 phosphorylation in the *Mecp2* transgenic mice. However, whether phosphorylation contributes to the observed behavioral phenotypes remains a topic for further investigation.

A list of pivotal signaling molecules related to lupus were investigated. BDNF serves as a target gene for MeCP2, and the plasma BDNF levels are significantly increased in SLE patients (67). Moreover, intrathecal albumin is increased in patients with CNS lupus (68), and it is an indicator of BBB damage in NPSLE patients (69). Furthermore, the intrathecal levels of GFAP are increased in SLE patients even in the absence of overt CNS disease (70). Consistent with these studies, our findings revealed intriguing elevations of BDNF, GFAP, and albumin in the brain of transgenic mice, similar to the observations of MRL/*lpr* mice. Besides, MeCP2 recruits the Sin3-histone deacetylase complex to promoters by binding methylated DNA through its MBD region, leading to transcriptional repression (71). In our study, both Sin3A and its phosphorylated form were increased in the MRL/*lpr* and B6.*Mecp2^Tg1^* mice, while the phosphorylation of CREB was slightly decreased compared to B6. Moreover, mTOR, NLRP3, and CD171 (L1CAM) are signaling molecules involved in the pathogenesis of lupus (72–74); interestingly, their expression and/or phosphorylation were influenced in the *Mecp2* transgenic mice.

In summary, the B6.*Mecp2^Tg1^* mice manifest a wide range of autoimmune phenotypes and neuropsychiatric characteristics akin to those observed in individuals with NPSLE, indicating its promise as a novel murine model for lupus investigations. Additionally, the model’s susceptibility to MeCP2 modulation via antisense oligonucleotides underscores its utility as an effective platform for assessing potential therapeutic strategies aimed at regulating MeCP2 levels. However, there are several limitations to consider in this study. Firstly, sex differences were observed in the B6. For *Mecp2^Tg1^* mice, the small sample size warrants the utilization of larger sample size to comprehensively explore the impact of MeCP2 expression on gender bias. Secondly, it is important to note that certain individuals with abnormal MeCP2 expression may induce hyperexcitability, resulting in neuronal activity aberrations. Therefore, MeCP2 overexpression and brain inflammation may concurrently contribute to the neuropsychiatric irregularities, yet the direct influence of MeCP2 on neurons and its potential contribution to autoimmune needs ascertainment. Finally, a comprehensive investigation involving human subjects is required to determine whether individuals harboring *Mecp2* polymorphisms exhibit an elevated susceptibility to developing NPSLE.

## Data availability statement

The original contributions presented in the study are included in the article/**Supplementary Material**. Further inquiries can be directed to the corresponding author.

## Ethics statement

The animal study was approved by Institutional Animal Care and Use Committee (IACUC) at the University of Houston. The study was conducted in accordance with the local legislation and institutional requirements.

## Author contributions

YL: Data curation, Formal analysis, Investigation, Methodology, Writing – original draft, Writing – review & editing. SZ: Data curation, Investigation, Methodology, Writing – review & editing. CT: Data curation, Formal analysis, Writing – review & editing. BY: Investigation, Writing – review & editing. FA: Methodology, Writing – review & editing. ZR: Methodology, Resources, Writing – review & editing. CM: Data curation, Writing – review & editing. SS: Methodology, Resources, Writing – review & editing. TW: Conceptualization, Funding acquisition, Project administration, Resources, Supervision, Writing – review & editing.
