# Supplementary material for: Autoimmune and neuropsychiatric phenotypes in a Mecp2 transgenic mouse model on C57BL/6 background
Source: Front Immunol. 2024 Mar 8;15:1370254. doi: 10.3389/fimmu.2024.1370254 (PMC10960363; doi:10.3389/fimmu.2024.1370254)
Supplement: Supplementary file 1 [file DataSheet_1.pdf]

Supplementary Table 1. List of antigens used in the antigen array.

**Antigen**	**Abbreviations in HeatMap**	**Gene ID#**
ADAMTS like 5	ADAMTSL5	339366
Aggrecan	ACAN	176
Annexin A11	ANXA11	311
Azurocidin 1	AZU1	566
β2-glycoprotein I	β2-GPI	350
TNF superfamily member 13b	TNFSF13B	10673
Branched chain 2-oxo acid dehydrogenase complex E2	BCOADC-E2	1629
Bactericidal permeability increasing protein	BPI	671
Bovine serum albumin	BSA	213
Complement C3	C3	718
Mucin 1	MUC1	4582
Carcinoembryonic antigen	CEA	1048
Cardiac muscle troponin T	cTnT	7139
Cathepsin D	CTSD	1509
Cathepsin G	CTSG	1511
Centromere protein B	CENPB	1059
Chondroitin sulphate	CS	N/A
C-Myc	C-Myc	4609
Type I Collagen	Collagen I	1277
Cyclin B1	Cyclin B1	891
Cytochrome c	Cyt-C	54205
Cytokeratin 6	CK 6	3853
Cytokeratin-14	CK 14	3861
Cytokeratin 17	CK 17	3872
Double stranded DNA	ds-DNA	N/A
Desmoglein 1	DSG1	1828
Glycyl-tRNA synthetase	EJ	2617
Elastase	Elastase	1991
Elastin	Elastin	2006
Endostatin	Endostatin	80781
Fibrinogen	FGN	522039
Fibronectin	FN	2335
Gastric Parietal Cell	GPC	495
Glycoprotein 210	Gp210	35512
Glomerular basement membrane	GBM	N/A
Hemocyanin, succinylated	Succinylated Hc	N/A
Heparan sulfate sodium salt	HS	N/A
Heparin sodium salt	Heparin	N/A
Heterogeneous nuclear ribonucleoprotein A1	hnRNPA1	3178
Histone H3	H3	8350
Histone H4	H4	8370

High mobility group box 1	HMGB1	3146
Helicobacter pylori	H. pylori	N/A
Heat shock protein 27kd	Hsp 27	3315
Heat shock protein 60kd	Hsp 60	3329
Heat shock protein 70kd	Hsp 70	3303
Heat shock protein 90kd	Hsp 90	3320
Hyaluronic acid sodium salt	HA	N/A
Insulin	Insulin	N/A
Intrinsic factor	IF	2694
Ku(p70/p80)	Ku (p70/p80)	N/A
Laminin	LM	16772
Lactoferrin	LTF	613079
Formiminotransferase Cyclodeaminase	FTCD	10841
Lysozyme	LZ	4069
Mitochondrial 2-oxo acid dehydrogenase complexes	M2	N/A
Mitochondrial Antigen	MA	N/A
Melanoma Differentiation-Associated Protein 5	MDA5	64135
Myelin Basic Protein	MBP	4155
Myeloperoxidase	MPO	4353
Myosin Heavy Chain	MHC	79784
Nucleoporin 62	Nup 62	23636
Threonyl tRNA synthetase 7	TRS 7	N/A
Threonyl tRNA Synthetase 12	TRS 12	N/A
Polymyositis/scleroderma 100	PM/Scl-100	5394
Proteinase 3	PR3	5657
Proteoglycan	PG	N/A
Ribosomal phosphoprotein P0	RPLP0	6175
Ribosomal phosphoprotein P1	RPLP1	6176
Ribosomal phosphoprotein P2	RPLP2	6181
Bovine RNP/Sm ribonucleoprotein complex	Sm/RNP	N/A
Squamous cell carcinoma antigen 1	SCCA1	6317
Squamous cell carcinoma antigen 2	SCCA2	6318
DNA Topoisomerase I	TOP1	7150
Sp100 Nuclear Antigen	SP100	6672
Survivin	Survivin	332
Thyroglobulin	Tg	7038
Tissue transglutaminase	tTG	7052
Yeast from Saccharomyces cerevisiae Type I	YSC1	N/A
Small nuclear ribonucleoprotein 68/70 kDa	U1-snRNP 68/70	6625
U1 small nuclear ribonucleoprotein A	U1-snRNP A	6626
U1 small nuclear ribonucleoprotein BB	U1-snRNP BB	6628
U1 small nuclear ribonucleoprotein C	U1-snRNP C	6631
Vitronectin	VTN	7448

Immunoglobulin G	IgG	N/A

**ORF**	**Vendor**	**Catalog#**
NP_998769.2	Novus	NBP1-93438PEP
NP_001126.3	Sigma	A1960
NP_001148.1	Abcam	ab101050
NP_001691.1	Arodia	ATA01-02
NP_000033.2	Diarect Ag	14900
NP_006564.1	Abcam	ab78840
NP_001909.3	Diarect Ag	17700
NP_001716.2	Arodia	ATB01-02
NP_000468.1	Sigma	A3059
NP_000055.2	BBI Solutions	P150-0
NP_001018016.1	Lee Biosolutions	151-53
NP_001278413.1	Lee Biosolutions	151-09
NP_000355.2	Prospec	PRO-342
NP_001900.1	Lee Biosolutions	186-08
NP_001902.1	Arodia	ATC01-02
NP_001801.1	Diarect Ag	12500
N/A	Sigma	C4384
NP_002458.2	RayBiotech	230-00580-50
NP_000079.2	Chondrex	1002
NP_114172.1	Abcam	ab128445
NP_061820.1	Sigma	C2037
NP_005545	antibodies	ABIN2712834
NP_000517.2	Abcam	ab73637-20
NP_000413.1	RayBiotech	230-00561-50
N/A	Sigma	D1501
NP_001933.2	Abnova	H00001828-PO1
NP_002038.2	Diarect Ag	11100
NP_001963.1	Sigma	E8140-1UN
NP_000492.2	Sigma	E1625
NP_085059.2	Sigma	SRP3031-100UG
NP_001028798.1	Sigma	F8630
NP_997647.1	MyBiosource	MBS635038
NP_000695.2	Arodia	ATP01-02
NP_610184.2	Diarect Ag	19000
N/A	Diarect Ag	16800
N/A	Sigma	H5654
N/A	Sigma	H7640
N/A	Sigma	H4784
NP_002127.1	Absci	AP70400
NP_003520.1	Roche	1034-758
NP_003539.1	Biolabs	M2504S

NP_002119.1	Chondrex	9050
N/A	MyBiosource	MBS537591
NP_001531.1	Abcam	ab48740
NP_002147.2	ENZO	ADI-SPP-540-050
NP_005336.3	ENZO	ADI-ESP-555-D
NP_005339.3	ENZO	ADI-SPP-770-D
N/A	Sigma	H7630
N/A	Sigma	91077C-100MG
NP_005133.2	Diarect Ag	16700
N/A	Diarect Ag	17300
NP_032506.2	Sigma	L2020
NP_001027487.1	Arodia	ATL02-02
NP_006648.1	Diarect Ag	13700
NP_000230.1	Sigma	L1667-1G
N/A	Diarect Ag	18000
N/A	Arodia	ATM02-02
NP_071451.2	Diarect Ag	30000
NP_002376.1	Signal Chem	M42-51N
NP_000241.1	BBI Solutions	P257-5
NP_079005.3	Sigma	M7659
NP_036478.2	Diarect Ag	19400
N/A	Diarect Ag	15600
N/A	Diarect Ag	15700
NP_002676.1	Diarect Ag	16000
NP_002768.3	Diarect Ag	18600
N/A	Sigma	P5864
NP_000993.1	Diarect Ag	14100
NP_000994.1	Diarect Ag	14200
NP_000995.1	Diarect Ag	14300
N/A	Diarect Ag	11600
NP_008850.1	Prospec	PRO-2198
NP_002965.1	RayBiotech	230-30014-50
NP_003277.1	Diarect Ag	12400
NP_003104.2	Diarect Ag	18900
O15392	Abcam	ab87202
NP_003226.4	Diarect Ag	12200
NP_004604.2	Diarect Ag	15200
N/A	Sigma	YSC1
NP_003080.2	Diarect Ag	13000
NP_004587.1	Diarect Ag	13100
NP_003082.1	Diarect Ag	13300
NP_003084.1	Diarect Ag	13200
NP_000629.3	Sigma	V8379

N/A	BBI Solutions	P525-3

Supplementary Table 2. Antibody list for western blot.

**Ab name**	**Vendor**	**Catalog#**	**Dilution**
Recombinant Anti-MeCP2 antibody [EPR23201-3]	Abcam	ab253197	1:1000
MeCP2 (phospho S421)	Abcam	ab254050	1:1000
Phospho-MeCP2(S80) Antibody	Abcepta	AP3595a-ev	1:500
Anti-BDNF antibody [EPR1292]	Abcam	ab108319	1:2000
GFAP (D1F4Q) XP	CST	12389s	1:1000
Recombinant Anti-Albumin antibody	Abcam	ab207327	1:2000
mSin3A Antibody (G-11)	Santa Cruz	sc-5299	1:500
Phospho-SIN3A (Ser832) Polyclonal Antibody	Invitrogen	PA5-105063	1:500
CREB (48H2) Rabbit mAb	CST	9197S	1:1000
Phospho-CREB (Ser133)	CST	9198S	1:1000
mTOR (7C10) Rabbit mAb	CST	2983S	1:1000
Phospho-mTOR (Ser2481) Antibody	CST	2974S	1:1000
Phospho-mTOR (Ser2448) (D9C2) XP(R) Rabbit mAb	CST	5536S	1:1000
NLRP3 (D4D8T) Rabbit mAb	CST	15101S	1:1000
Phospho-NLRP3 (Ser295) Polyclonal Antibody	Invitrogen	PA5-105071	1:500
CD171 Monoclonal Antibody	Invitrogen	MA5-14140	1:200
Phospho-CD171 (Ser1181) Polyclonal Antibody	Invitrogen	PA5-38442	1:500
TBLR1 Antibody	Novus	NB600-270	1:2000
RASGEF1B Polyclonal Antibody	Invitrogen	PA5-104060	1:1000
Recombinant Anti-PPP2R2B antibody	Abcam	ab157461	1:2000
BANK1 Antibody (F-8)	Santa Cruz	sc-393611	1:500
TREX1 Antibody	CST	76892S	1:1000
Thrombospondin-1 (D7E5F) Rabbit mAb	CST	37879S	1:1000
β-Actin (D6A8) Rabbit mAb	CST	8457S	1:1000
GAPDH (14C10) Rabbit mAb	CST	2118s	1:1000

Supplementary Table 3. Comparasion of each autoantibody level in different mouse groups and its correlation with ds

Antigen	*P*- value compared to B6 (two-tailed)			Correlation to anti-dsDNA antibody		
	MRL/*lpr*	B6.*Mecp2* ^{Tg1} Female	B6.*Mecp2* ^{Tg1} Male	r^2	*P*- value (two-tailed)	*P*- value summary
ADAMTSL5	0.0691	0.0356	0.0869	0.3126	0.0588	ns
ACAN	0.0032	0.1386	0.2591	0.7753	**0.0002**	***
ANXA11	0.0195	0.0014	0.5925	0.02591	0.6173	ns
AZU1	0.0052	0.0976	0.7328	0.5495	**0.0058**	**
β2-GPI	0.0000	0.2179	0.2351	0.4408	**0.0185**	*
TNFSF13B	0.1295	0.5957	0.8752	0.8292	**<0.0001**	****
BCOADC-E2	0.0381	0.0582	0.2267	0.3971	**0.0281**	*
BPI	0.0622	0.0571	0.1132	0.3795	**0.0329**	*
BSA	0.2302	0.9504	0.1897	0.0908	0.3412	ns
C3	0.0211	0.1876	0.3914	0.4532	**0.0164**	*
MUC1	0.2873	0.1958	0.9458	0.1614	0.1954	ns
CEA	0.0632	0.0532	0.4378	0.5304	**0.0072**	**
cTnT	0.0277	0.0432	0.1438	0.3883	**0.0304**	*
CTSD	0.2477	0.2450	0.4935	0.1146	0.2817	ns
CTSG	0.0093	0.0555	0.1216	0.7368	**0.0004**	***
CENPB	0.0140	0.1555	0.5251	0.6449	**0.0017**	**
CS	0.0122	0.5024	0.2253	0.7393	**0.0003**	***
C-Myc	0.0254	0.0822	0.3879	0.1677	0.1862	ns
Collagen I	0.8393	0.0124	0.1105	0.02776	0.6047	ns
Cyclin B1	0.0312	0.0132	0.0191	0.3467	**0.044**	*
Cyt-C	0.0608	0.1359	0.2843	0.2525	0.0959	ns
CK 6	0.0373	0.2100	0.2334	0.8928	**<0.0001**	****
CK 14	0.1075	0.1682	0.1955	0.7179	**0.0005**	***
CK 17	0.0003	0.0394	0.0499	0.7927	**0.0001**	***
ds-DNA	0.0506	0.3769	0.3946	-	-	-
DSG1	0.0318	0.1805	0.8252	0.7249	**0.0004**	***
EJ	0.0017	0.0156	0.1402	0.5518	**0.0056**	**
Elastase	0.0522	0.2126	0.9327	0.5596	**0.0051**	**
Elastin	0.0726	0.1768	0.9726	0.5507	**0.0057**	**
Endostatin	0.0666	0.0170	0.9705	0.4863	**0.0117**	*
FGN	0.0008	0.8734	0.5515	0.7302	**0.0004**	***
FN	0.0474	0.0877	0.1900	0.6306	**0.002**	**
GPC	0.0888	0.0160	0.1229	0.2861	0.0732	ns
Gp210	0.0236	0.1025	0.3144	0.4206	**0.0225**	*
GBM	0.6359	0.0729	0.2515	0.04418	0.512	ns
Succinylated H(	0.0818	0.1098	0.3614	0.6088	**0.0028**	**
HS	0.0482	0.3245	0.6454	0.3493	**0.043**	*
Heparin	0.2565	0.0286	0.0494	0.1964	0.149	ns

hnRNPA1	0.8881	0.0362	0.1153	5.75E-05	0.9813	ns
H3	0.0121	0.1478	0.2117	0.5605	**0.0051**	**
H4	0.0087	0.1961	0.4226	0.668	**0.0012**	**
HMGB1	0.0776	0.8291	0.6184	0.7164	**0.0005**	***
H. pylori	0.0170	0.2541	0.8732	0.6178	**0.0024**	**
Hsp 27	0.0722	0.0669	0.2921	0.5596	**0.0051**	**
Hsp 60	0.0686	0.8082	0.7030	0.3699	**0.0359**	*
Hsp 70	0.0302	0.3585	0.4034	0.529	**0.0073**	**
Hsp 90	0.0850	0.0549	0.5475	0.4086	**0.0252**	*
HA	0.0356	0.0030	0.0009	0.4945	**0.0107**	*
Insulin	0.0812	0.0034	0.0405	0.2188	0.1251	ns
IF	0.0597	0.0801	0.0563	0.1832	0.1651	ns
Ku (p70/p80)	0.0023	0.0297	0.4669	0.628	**0.0021**	**
LM	0.0134	0.0043	0.9585	0.4878	**0.0115**	*
LTF	0.0110	0.1809	0.8585	0.4917	**0.0111**	*
FTCD	0.0456	0.0900	0.3973	0.2649	0.0869	ns
LZ	0.1671	0.6194	0.1123	0.4027	**0.0266**	*
M2	0.0051	0.0442	0.2953	0.5236	**0.0078**	**
MA	0.9937	0.8386	0.0023	0.001653	0.9002	ns
MDA5	0.0669	0.4875	0.3674	0.3522	**0.0419**	*
MBP	0.1849	0.5217	0.5311	0.3913	**0.0296**	*
MPO	0.0282	0.2671	0.9742	0.4077	**0.0254**	*
MHC	0.0028	0.0533	0.0742	0.3718	**0.0353**	*
Nup 62	0.2957	0.2184	0.0942	0.002406	0.8797	ns
TRS 7	0.0030	0.0130	0.4319	0.7306	**0.0004**	***
TRS 12	0.2115	0.5494	0.5542	0.04869	0.4907	ns
PM/Scl-100	0.0046	0.7988	0.4167	0.5893	**0.0036**	**
PR3	0.1157	0.6122	0.3396	0.5258	**0.0076**	**
PG	0.0110	0.2220	0.8209	0.8627	**<0.0001**	****
RPLP0	0.0003	0.0362	0.2415	0.777	**0.0002**	***
RPLP1	0.0000	0.0287	0.0824	0.6657	**0.0012**	**
RPLP2	0.0441	0.0262	0.6048	0.4976	**0.0104**	*
Sm/RNP	0.0366	0.5500	0.1563	0.562	**0.005**	**
SCCA1	0.6782	0.8960	0.4067	0.0229	0.6388	ns
SCCA2	0.8424	0.9922	0.8468	0.07207	0.3989	ns
TOP1	0.0043	0.1356	0.9265	0.659	**0.0013**	**
SP100	0.0280	0.7926	0.0207	0.1434	0.2249	ns
Survivin	0.0002	0.8308	0.0018	0.3183	0.056	ns
Tg	0.0090	0.6766	0.0209	0.193	0.153	ns
tTG	0.0008	0.0380	0.8608	0.6763	**0.001**	**
YSC1	0.0804	0.5031	0.1430	0.4155	**0.0236**	*
U1-snRNP 68/7	0.0517	0.4393	0.5509	0.621	**0.0023**	**
U1-snRNP A	0.0632	0.0290	0.4528	0.1919	0.1544	ns

U1-snRNP BB	0.1019	0.1470	0.5231	0.4136	**0.0241**	*
U1-snRNP C	0.0906	0.4091	0.1814	0.4385	**0.019**	*
VTN	0.0036	0.2052	0.9409	0.6168	**0.0025**	**
IgG	0.1147	0.9570	0.3575	0.2492	0.0985	ns

n = 3 per group. *, P < 0.05, **, P < 0.01, ***, P < 0.001, ****, P < 0.0001. Red marked *P* -value represents significantly changed antibody level in that mouse group compared to B6. Bolded *P* -values represent antibodies have important correlation with the anti-dsDNA antibody level.

sDNA.
