# Supplementary material for: Autoimmune and neuropsychiatric phenotypes in a Mecp2 transgenic mouse model on C57BL/6 background
Source: Front Immunol. 2024 Mar 8;15:1370254. doi: 10.3389/fimmu.2024.1370254 (PMC10960363; doi:10.3389/fimmu.2024.1370254)
Supplement: Supplementary file 2 [file DataSheet_2.docx]

Supplementary Materials

**
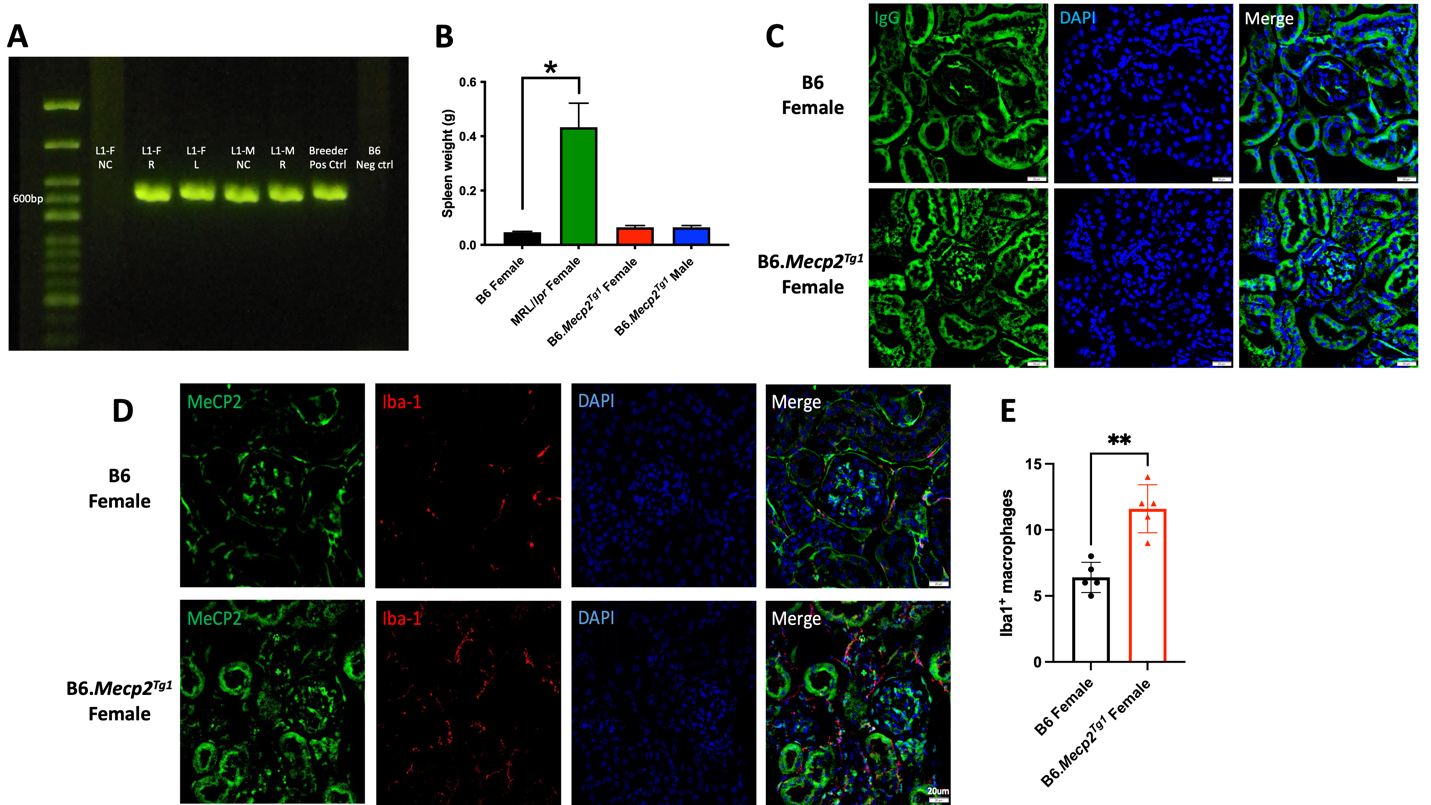
**

**Supplementary Figure 1.** IgG deposition and macrophage infiltration in the kidney of female B6.*Mecp2^Tg1^* mice. (A) Example of genotyping result from the first batch litters generated by the B6.*Mecp2^Tg1^* breeders. The first lane shows DNA ladder, lanes 2-6 are added with DNA samples from the litters, and lane 7 and lane 8 are added with DNA samples from one breeder as a positive control and B6 as a negative control, respectively. Target *Mecp2* gene is observed at 600bp. (B) Measurement of spleen weight for all mice groups. Mice are sacrificed at 17-weeks-old, n = 3 per group. *, *P* < 0.05. (C) Immunofluorescence images of IgG in the glomerulus area of B6 and female *Mecp2* transgenic mice. Green, IgG; Blue, DAPI labeled nuclei; the third lane, images merged by the previous two lanes. Scale bar: 20 μm. (D) Macrophage infiltration in the kidney of B6 and female *Mecp2* transgenic mice. Green, MeCP2; red, Iba-1 labeled macrophages; blue, DAPI labeled nuclei; the fourth lane, images merged by the previous three lanes. Scale bar: 20 μm.

(E) Statistical analysis of Iba1^+^ macrophages in the kidney glomerulus area in female B6 control and B6.*Mecp2^Tg1^* mice. Mice were sacrificed at 17 weeks of age, with n = 5 per group. **, *P* < 0.01.


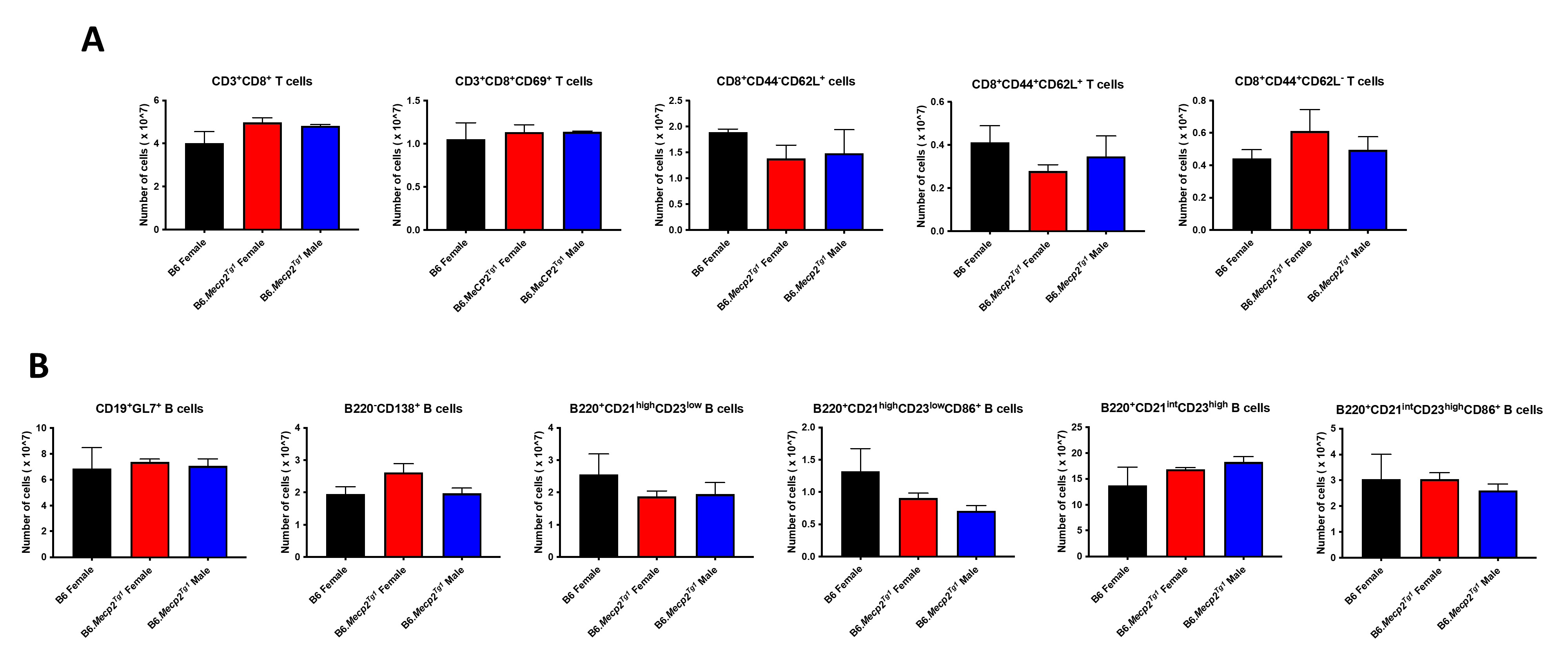


**Supplementary Figure 2.** Cell number changes of CD3^+^CD8^+^ T cell subsets and the B cell subsets in the B6.*Mecp2^Tg1^* mice. (A) Cell number changes of CD3^+^CD8^+^ and its activation form, as well as the changes of its subsets CD8^+^CD44^-^CD62L^+^ naïve T cells, CD8^+^CD44^+^CD62L^+^ central memory T cells, and CD8^+^CD44^+^CD62L^-^ effector memory T cells. (B) Cell number changes of CD19^+^GL7^+^ germinal center cells, B220^-^CD138^+^ plasma B cells, as well as CD21^int^CD23^high^ follicular B cells, CD21^high^CD23^low^ marginal zone B cells and their activation forms. n = 3 per group.


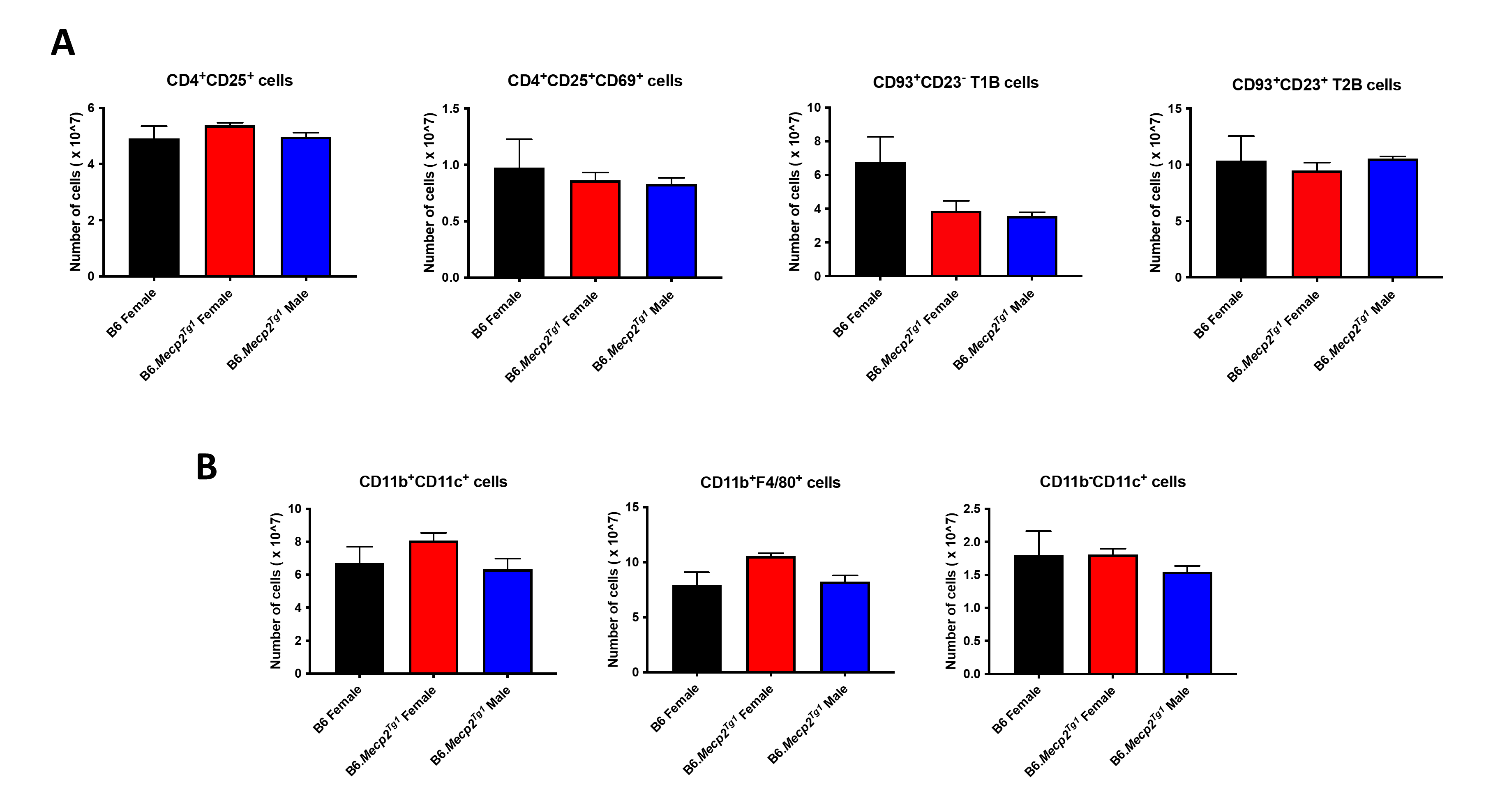


**Supplementary Figure 3.** Cell number changes of regulatory T cells, transitional T cells and myeloid cell subsets in the B6.*Mecp2^Tg1^* mice compared to B6. (A) Cell number changes of CD4^+^CD25^+^ regulatory T cells, its CD69^+^ labeled activation form, CD93^+^CD23^-^ T1B and CD93^+^CD23^+^ T2B cells. (B) Cell number changes of CD11c^+^CD11b^+^ dendritic cells, CD11c^-^CD11b^+^ dendritic cells, and CD11b^+^F4/80^+^ macrophages. n = 3 per group.


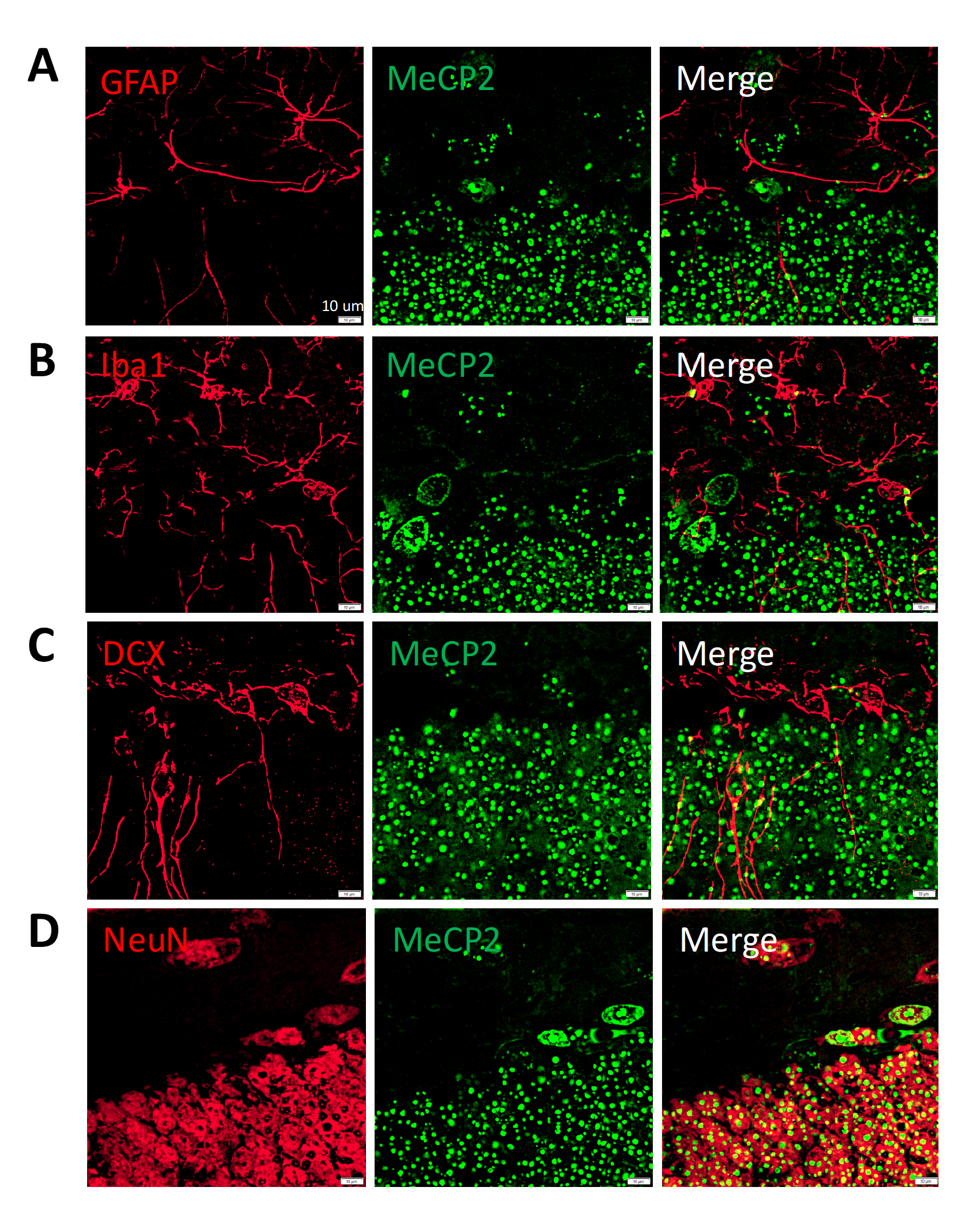


**Supplementary Figure 4.** MeCP2 is mainly expressed in mature neurons. (A-D) Double staining of green MeCP2 and red astrocyte marker GFAP, microglia marker Iba-1, newborn neuron marker DCX, and mature neuron marker NeuN in the subgranular zone. Third lane, merged images. Scale bar: 10 μm.

**
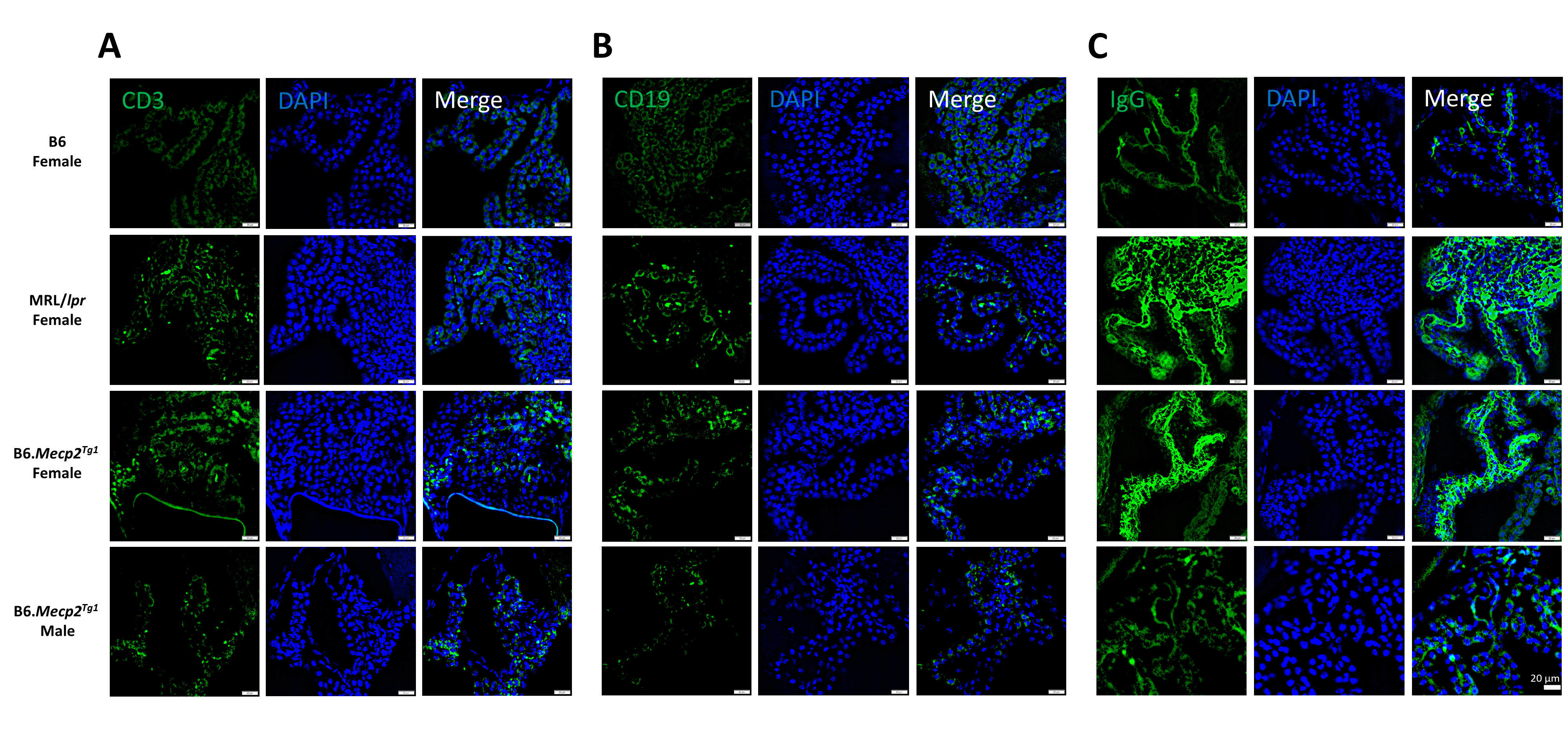
**

**Supplementary Figure 5.** Immune cell enrichment and IgG deposition in the choroid plexus. (A-B) Immunofluorescence staining of T cells and B cells in all mice groups. Green, FITC labeled CD3^+^ T cells or CD19^+^ B cells; blue, DAPI labeled nuclei; third lane, image merged by the previous lanes. (C) Immunofluorescence staining of anti-mouse IgG. Green, FITC labeled IgG; blue, DAPI labeled nuclei; third lane, image merged by the previous lanes. Scale bar: 20 μm.


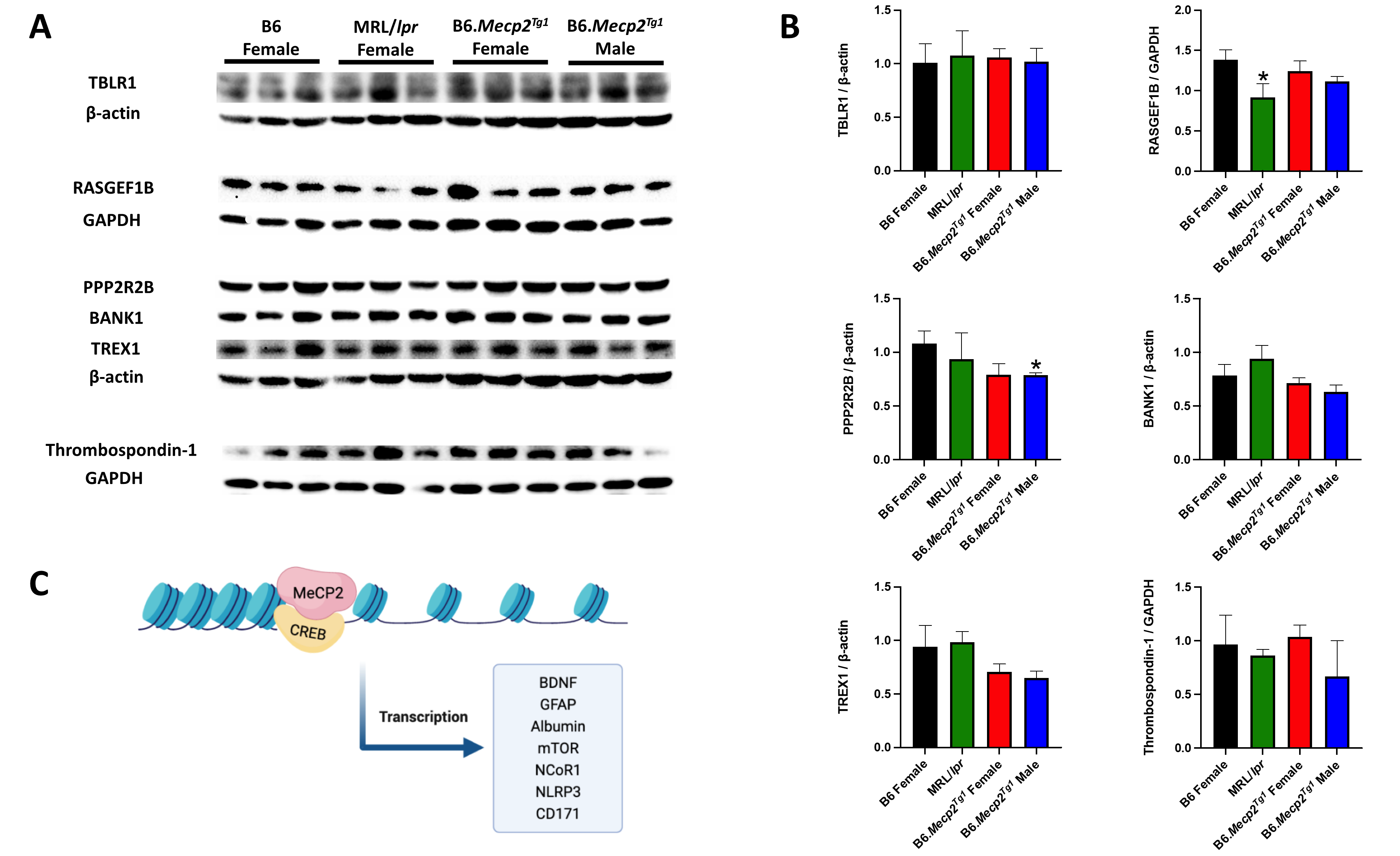


**Supplementary Figure 6.** Expression of signaling molecules involved in lupus pathogenesis. (A-B) Brain expression of TBLR1, RASGEF1B, PPP2R2B, BANK1, TREX1, and Thrombospondin-1 were measured through western blot and the results were normalized through β-actin or GAPDH. n = 3 per group. *, *P* < 0.05. (C) Summary of elevated signaling molecules in the MeCP2 overexpression mice.
